# Phenotypic profiling of neutrophils in acute *Clostridioides difficile* infection identifies a TNF-induced activation signature associated with epithelial damage

**DOI:** 10.1080/19490976.2026.2659430

**Published:** 2026-04-23

**Authors:** Alexander Huber, Ann Mathew, Faiza Khondokar, Divya Sharma, Anindita Mukherjee, Dolly K. Khona, Jucy Gabriel, Shinsmon Jose, Rajat Madan

**Affiliations:** a Infectious Disease Division, Department of Medicine, University of Cincinnati, Cincinnati, OH, USA; b Division of Immunobiology, Cincinnati Children's Hospital Medical Center, Cincinnati, OH, USA; c Department of Pathology and Laboratory Medicine, University of Cincinnati, Cincinnati, OH, USA; d Division of Infectious Diseases, Department of Medicine, University of Arizona, Tucson, AZ, USA; e Veterans Affairs Medical Center, Tucson, AZ, USA

**Keywords:** Neutrophil, *Clostridioides difficile* infection, innate immunity, TNF, intestinal epithelium

## Abstract

Neutrophils are key first responders in the host response to *Clostridioides difficile* infection (CDI). Although a high number of tissue and blood neutrophils clearly correlates with adverse outcomes in CDI patients, their functional role remains poorly defined. Using murine (CMT-93) and human (Caco-2) intestinal epithelial cell (IEC) lines co-cultured with neutrophils and a pre-clinical mouse model of CDI, we show that activated neutrophils exacerbate *C. difficile*-induced IEC injury. To identify neutrophil subtypes and biological pathways that contribute to CDI-associated IEC damage, we utilized single-cell RNAseq (scRNAseq) to generate the first transcriptomic atlas of murine bone marrow, blood, and colonic neutrophils in acute CDI. Our analyses identified a novel neutrophil population in the colonic lamina propria, “cNeu3,” which exhibited high expression of genes associated with neutrophil activation and degranulation. Informed by cNeu3 signature genes from the scRNAseq dataset, we validated the presence of these neutrophils in mouse colon using flow cytometry (CD11b^hi^CD63^hi^IL-1β^hi^MIP-1α^hi^MALT1^hi^ cells). We found that their abundance correlated with increased epithelial damage. Tumor Necrosis Factor (TNF) was sufficient to polarize mouse bone marrow neutrophils to a cNeu3 phenotype *in vitro*. Finally, TNF-primed human neutrophils worsened *C. difficile* toxin-induced IEC damage, which was improved when neutrophil degranulation was blocked. Collectively, our data provide novel insights into neutrophil-mediated pathology during CDI.

## Introduction

Polymorphonuclear leukocytes, or neutrophils, are critical first responders to infectious insults, particularly those caused by bacteria.^1^^,^^2^ Neutrophils exert antimicrobial effects by phagocytosing invading pathogens, degranulating an arsenal of cytotoxic granule proteins, and releasing neutrophil extracellular traps (NETs).^1^^,^^2^ However, uncontrolled or inappropriate neutrophil activation can lead to collateral tissue damage and a worsening of pathology in several diseases, such as SARS-CoV-2 infection, chronic obstructive pulmonary disease, and colitis.^2-5^ *Clostridioides difficile* infection (CDI)-induced colitis is a leading cause of nosocomial diarrhea in the U.S. and is an urgent antibiotic-resistant threat.^6-8^ Although *C. difficile* toxins drive initial intestinal epithelial cell (IEC) damage, multiple studies have linked key components of host innate immune response—such as cytokines, neutrophils, and neutrophil-derived products—to worse disease outcomes.^9-14^ While neutrophils are clearly implicated in shaping clinical outcomes, their direct role in regulating tissue injury observed in *C. difficile* colitis has not been studied.

Traditionally, neutrophils were considered to be relatively homogeneous and transcriptomically inactive cells, and subpopulations were mostly defined based on nuclear morphology, maturation status, cell surface receptor expression, and release of effector molecules.^2^ However, more recently, this concept has been challenged, and high-dimensional analysis techniques like single-cell RNA sequencing (scRNAseq) and proteomic profiling have shown that neutrophils are clearly heterogeneous—they exist on a developmental trajectory which changes based on systemic and tissue microenvironmental cues.^15-18^ Further, stress-driven myelopoiesis drastically changes neutrophil dynamics from a homeostatic state, resulting in the expansion and development of new populations represented by different transcriptomic and proteomic signatures.^18-20^

To gain a deeper understanding of how neutrophils impact CDI pathogenesis, we examined the role of these cells in mice with CDI and in mouse and human neutrophil-IEC co-cultures. Our data reveal that neutrophils are key pathogenic determinants in exacerbating IEC barrier damage during the acute phase of CDI (i.e., on day one to two after infection). Leveraging the power of scRNAseq, we established a transcriptomic atlas of bone marrow, blood, and colonic neutrophils in acute CDI. We demonstrate that a large majority of neutrophils recruited to the colonic lamina propria exhibit high expression of genes associated with activation and degranulation, and similar clusters of hyperactive neutrophils were present in bone marrow and blood of mice with CDI. Directed by the transcriptomic signature of these CDI-induced clusters, we identified that intracellular IL-1β, MIP-1α, and MALT1, and cell surface CD11b and CD63 are key flow cytometry markers for a hyperactivated neutrophil state that is associated with increased barrier damage. Utilizing a wide range of transcriptomic analyses and complementary *in vitro* tissue culture experiments, our data suggest that Tumor Necrosis Factor (TNF) induces this distinct neutrophil phenotype. scRNAseq pointed to neutrophil degranulation as one of the top CDI-induced pathways in bone marrow, blood, and colon neutrophils, and blocking degranulation in TNF-primed human neutrophils with an exocytosis inhibitor reduced IEC damage caused by these cells *in vitro*.

## Materials and methods

### Mice

C57BL/6 wildtype mice (https://www.jax.org/strain/000664), MRP8-Cre^+^ mice (B6.Cg-Tg*(S100A8-cre,-EGFP)1Ilw*/J; https://www.jax.org/strain/021614), and ROSA-iDTR^KI^ (C57BL/6-*Gt(ROSA)26Sor*^*tm1(HBEGF)Awai*^/J; https://www.jax.org/strain/007900) mice were purchased from The Jackson Laboratory. iDTR^+/+^Cre^+^ mice and littermate iDTR^+/+^Cre^−^ were generated by crossing MRP8-Cre^+^ mice with ROSA-iDTR^KI^, as previously described.^21^ Genotypes of the animals were confirmed by the Jackson Laboratory's standard PCR assays (protocols 22392 and 29915). All mice were maintained at the animal facilities of the University of Cincinnati and the Cincinnati VA Medical Center, accredited by the American Association for Accreditation of Laboratory Animal Care (Frederick, MD), under protocols approved by the Institutional Animal Care and Use Committee (IACUC).

### Mouse model of CDI

Eight to thirteen-week-old age- and sex-matched C57BL/6 wildtype (WT), iDTR^+/+^Cre^+^, and littermate iDTR^+/+^Cre^−^ control mice (all housed under pathogen-free conditions) were pretreated with antibiotics to induce gut dysbiosis prior to *C. difficile* challenge. Dysbiosis was induced in mice by using one of two previously defined protocols, either: (a) 0.5 mg/ml cefoperazone in drinking water for 5 d followed by 2 d of water without antibiotics prior to *C. difficile* challenge; or (b) a mixture of vancomycin (0.045 mg/ml), gentamicin (0.035 mg/ml), kanamycin (0.4 mg/ml), colistin (850 U/ml), and metronidazole (0.215 mg/ml) in drinking water for 3 d, followed by 2 d of water without antibiotics along with an intraperitoneal injection (i.p.) of clindamycin (10 mg/kg), the day before *C. difficile* challenge.^22-25^ Mice were orally gavaged with purified *C. difficile* spores (1 × 10^6^ M7404 spores/mouse) after antibiotic-induced gut dysbiosis.^22^^,^^26^^,^^27^ Infected mice were singly housed and monitored daily for weight loss and diarrhea.^28^^,^^29^ Diarrhea was scored from zero to three based on stool consistency (0 = normal formed stool pellet; 1 = soft discolored stool; 2 = watery stool/wet stained tail; 3 = mucus discharge/no stool with previous episode of diarrhea), as per previously published protocols.^24^ For neutrophil depletion, iDTR^+/+^Cre^+^ and iDTR^+/+^Cre^−^ mice received i.p. injections of diphtheria toxin (500 ng; Sigma-Aldrich) prior to infection, as previously described.^21^^,^^30^ On pre-specified days after infection, mice were euthanized by CO_2_ inhalation, and blood, tissue, and intra-cecal samples were collected for further processing. For the experiment where we examined the longer-term impact of neutrophil depletion, DT injection was given for 2 additional days at 24-hour intervals (i.e., on day 1 and day 2 after *C. difficile* challenge).

### Murine blood and lamina propria leukocyte isolation and flow cytometry

Blood was obtained by intracardiac puncture and collected in ethylenediaminetetraacetic acid microtainers. Cecum and proximal colon tissue were obtained for lamina propria leukocyte isolation. In brief, 100 μl of blood and ~2 cm of cecal and proximal colon tissue samples were used to obtain single-cell suspensions of blood and lamina propria cells, as previously described.^24^^,^^31^ These cells (~10^6^ cells/tube) were stained with a LIVE/DEAD™ Fixable Blue Dead Cell Stain Kit (Thermo Fisher/Invitrogen #L23105) and Fc receptors were blocked with anti-CD16/32 (BD). Cells were then stained with fluorescent marker-conjugated antibodies to Ly6G (BioLegend #563978), CD11b (BioLegend #101259), and CD63 (Miltenyi #130-133-972). For intracellular staining, fixed and permeabilized cells were stained with antibodies to MALT1 (Proteintech #11660-1-AP; secondary: BioLegend #406421), MIP-1α (Thermo Fisher #46-7532-82), and IL-1β (Thermo Fisher #25-7114-82). Flow cytometry was performed using Cytek Aurora spectral flow cytometer or BD FACS Accuri C6 flow cytometer equipped with CFlow Plus software (BD Biosciences). All flow cytometry analysis was done using FlowJo v10.10.0 (Treestar) with FlowAI.^32^

To define cNeu3-like neutrophils, we performed two complementary analyses of the flow cytometry data: unsupervised clustering using FlowSOM with UMAP for visualization and conventional flow cytometry gating. Both strategies are shown in Supp Figure 5. Each experiment was analyzed independently, and identical parameters were applied. First, live cells from each host were concatenated into a single file, and neutrophils were identified as CD11b⁺Ly6G⁺ cells (Supp Figure 5A). FlowSOM, an unsupervised clustering algorithm that groups cells based on marker expression profiles, was then paired with UMAP to identify and visualize distinct neutrophil states.^28^^,^^33^^,^^34^ Since FlowSOM clustering is sensitive to outliers,^33^ we limited upper intensity values of our selected markers (i.e., CD11b, MALT1, MIP1-α, IL-1β, CD63, and Ly6G) to the 99th percentile as a way to avoid outliers from disproportionately influencing clustering analyses. Using UMAP dimensional reduction and FlowSOM clustering on FlowJo software, we then defined cell clusters that exhibited high CD63 expression together with elevated IL-1β, CD11b, MALT1, and MIP-1α as cNeu3-like neutrophils (Supp Figure 5B-C). Complementary analyses using conventional gating in FlowJo were performed to identify cNeu3-like neutrophils. In this case, live CD11b⁺Ly6G⁺ cells (Supp Figure 5A) were plotted for CD11b and IL-1β expression and then colored based on the amount of surface CD63 expression (Supp Figure 5D). To standardize CD63 color display across independent experiments, the color scale was adjusted in FlowJo by setting the upper limit to the 99th percentile of CD63 expression and the lower limit to the 1st percentile of CD63 expression, as calculated in FlowJo. These same percentile limits were applied to each experiment. After applying these limits, a distinct population of cells that had high expression of IL-1β, CD11b, and CD63 became visible and were defined as cNeu3-like neutrophils (Supp Figure 5D). Of note, these cells had high MFI of MALT1 and MIP-1α (Supp Figure 5D). Concordance between cNeu3-like populations defined by FlowSOM and by conventional gating was evaluated within each experiment (Supp Figure 5E-F). FlowJo, GraphPad Prism, and pheatmap in R were used for visualization and downstream analysis.

### Spore preparation

*C. difficile* spores were inoculated in Columbia broth from glycerol stock and grown anaerobically at 37 °C. After 2–3 d, 1 ml of culture was inoculated into 40  ml Clospore medium^35^ and re-incubated at 37 °C in an anaerobic chamber for 5–7 d. Cultures were centrifuged (3200 × *g*, 20  min, 4 °C), washed three times in ice-cold sterile water to release spores, and resuspended in 1 ml DPBS. Spore numbers were quantified by plating 1:10 dilutions onto Cycloserine Cefoxitin Fructose Agar with Horse Blood and Taurocholate (CCFA-HT; Anaerobe Systems #AS-2136) and incubated in an anaerobic chamber for 24 hours prior to enumeration.

### Estimation of *C. difficile* pathogen burden and toxin

Cecal contents were weighed and homogenized in 1× PBS followed by 10-fold serial dilutions. Diluted contents were plated in duplicate inside an anaerobic chamber at 37 °C onto CCFA-HT; according to the manufacturer's instructions.^36^ After 24 hours, distinct colonies from ~10^3^–10^6^ dilutions were counted. Data were normalized to the weight of each sample and reported as CFU/g of cecal contents. Toxin levels were measured from the homogenized samples using *C. difficile* TOXA/B ELISA kit (TechLab #30397) as per manufacturer's instructions.

### Histopathology of cecal tissue sections

Cecal tissues were fixed in Bouin's solution (Sigma-Aldrich), dehydrated in ethanol, and embedded in paraffin. Sections (4 μm) were stained with H&E and histopathological scoring was performed on a 0–4 scale for three parameters: edema (graded by severity and extent of submucosal expansion), cellular infiltration (graded by degree and distribution of neutrophilic inflammation and tissue involvement), and epithelial damage (graded by extent of epithelial injury, pseudomembrane formation, or ulceration) (Further details in Supp Table 1).

### FITC-dextran assay

In experiments assessing intestinal permeability, mice were gavaged with 100 μl of FITC-dextran (25 mg/ml FD4; Sigma) 18 hours after challenge and 4 hours after food restriction. FD4 leakage was assessed by measuring fluorescence in plasma 6 hours later using a BioTek Synergy H1 Plate Reader.^37-39^

### Neutrophil isolation

Bone marrow cells were obtained by flushing femurs and tibiae with PBS + 2% fetal bovine serum, followed by red blood cell lysis with ACK buffer (Lonza #10-548E).^13^ Neutrophils were enriched using MACS MS columns (Miltenyi #130-097-658) and stained with antibodies for Ly6G (BioLegend #127654 or #563978), CD11b (BioLegend #101259 or Thermo Fisher #12-0112-82), and viability dye (Thermo Fisher #L10120).^13^ Healthy human blood samples were purchased and provided to us in a de-identified manner from Hoxworth Blood Center. Human neutrophils were isolated using the Direct Human Neutrophil Isolation Kit (StemCell Technologies #19666) and then stained for CD11b (BioLegend #301403) and CD66b (BioLegend #392903). Purity of isolated neutrophils was checked by doing flow cytometry on a BD FACS Accuri C6 (typical samples used for assays had >95% neutrophils).

### Transepithelial electrical resistance

Mouse (CMT-93; ATCC) or human (Caco-2; ATCC) intestinal epithelial cells were seeded and grown to confluence in 96-well E-plates (Agilent #300601010) until TEER values stabilized. Purified mouse or human neutrophils (2.5 × 10^5^ cells/well) along with other stimuli were added simultaneously to the apical portion of confluent IEC monolayers, and impedance was measured at 15-minute intervals for up to 72 hours using the xCELLigence system. During the course of experiments, cells were maintained at 37 °C in a humidified incubator with 5% CO_2_. The culture media used for these experiments were Dulbecco's Modified Eagle Medium (for CMT-93) or MEM (for Caco-2) with 20% fetal bovine serum and 1% penicillin/streptomycin. Concentration of various stimuli used is based on published literature and pilot studies done in our lab: *C. difficile* toxins A and B (5 ng/ml each; List Biologicals #152C and #155L),^40^ LPS (1 μg/ml; InvivoGen #tlrl-eblps),^41-43^ recombinant TNF (20 ng/ml; R&D #210-TA-020),^44^^,^^45^ and Nexinhib20 (10 μM; Tocris #6089).^46^

### *In vitro* TNF polarization of murine neutrophils

Isolated bone marrow neutrophils were stimulated with or without recombinant murine TNF (50 ng/ml; R&D #410-MT-010) for 2 hours in RPMI-1640 (Thermo Fisher) + 10% FBS. TNF concentration used for these experiments was based on published literature^47^^,^^48^ and pilot studies in our lab. Cells were then stained and analyzed by flow cytometry as detailed above.

### ELISA

TNF levels in cecal homogenates were quantified using an ELISA kit (BioLegend #430901) and normalized to total protein measured using a BCA Protein Assay Kit (Thermo Fisher #23225).

### Single-cell RNAseq

For the single-cell transcriptomics study, neutrophils were obtained from an uninfected male mouse representing homeostatic conditions (bone marrow and blood neutrophils) and a *C. difficile*-challenged male mouse (bone marrow, blood, and colon neutrophils). Both mice were 10–12 weeks old at the time of collection. Neutrophils were enriched using negative selection kits (as described above), and epithelial cells were further depleted using CD326 MicroBeads (Miltenyi #130-105-958). Neutrophil sorting buffer was supplemented with 1 U/µl RNase inhibitor (Millipore Sigma RNAINH-RO). Cell viability was confirmed to be >70% by trypan blue exclusion. Libraries were prepared using the Chromium NextGEM Single Cell 3' v3.1 kit (10× genomics) and sequenced on an Illumina platform. Data were aligned to the mm10 genome using Cell Ranger v7.0.0. Post-sequencing quality control was performed in R; see Supplementary Methods for a detailed description. Neutrophil annotations were refined using AUCell and SingleR with references from PanglaoDB, Tabula Muris Senis, and ImmGen.^49-52^

### Functional and trajectory analysis

Gene set enrichment analysis was performed using Metascape^53^ and Reactome via SeuratExtend.^54^ Immune response enrichment was performed using IREA https://www.immune-dictionary.org. Trajectory inference was done using CytoTRACE^55^ and Monocle3,^56-58^ with Neu1 designated as the root. LOESS regression was used in R to visualize TNF module scores over pseudotime.

### Scoring of neutrophil function

Gene modules (see Supp Table 2) were scored with AddModuleScore in Seurat. Cell cycle scoring was performed using CellCycleScoring with genes from Nestorowa et al.^59^

### Bulk RNA-seq analysis

DEGs between untreated and TNF-stimulated neutrophils were identified from GSE40548^60^ and GSE70068,^61^ using dplyr and readr to filter for significant changes and compute log₂ fold change-based rankings, retaining only protein-coding genes. Gene signature lists representing different neutrophil maturation states were obtained from GSE109467^62^ by analyzing raw counts with DESeq2, applying variance-stabilizing transformation, and performing marker gene identification by group comparison; top genes for each stage were combined with the most variable genes and visualized in an annotated heatmap using pheatmap. For GSE276395,^63^ genes upregulated in CDI patients were obtained by performing differential expression analysis using GEO2R (https://www.ncbi.nlm.nih.gov/geo/geo2r/), importing the results into R, filtering for significant changes, extracting gene symbols from the “gene_assignment” column, and selecting the top genes for downstream analyses.

### Visualization and statistical analysis

Data visualization was performed in GraphPad Prism v5.0 and R using Seurat, ggplot2, ScCustomize, ScPubR, and SeuratExtend.^54^^,^^64-67^ Graphics and illustrations were created using BioRender.com. Statistical analyses were conducted in GraphPad Prism v5.0 and R. Statistical tests were selected based on data distribution and variance. Normality was assessed using the Shapiro–Wilk test. For normally distributed data with unequal variances, an unpaired Student’s *t*-test with Welch's correction was used. For non-normally distributed data, a two-tailed Mann–Whitney test was applied. Correlations between non-normally distributed variables or variables without a linear relationship were assessed using Spearman’s rank correlation. For comparisons across more than two groups, a one-way ANOVA or a two-way ANOVA was used. Area under the curve (AUC) values for TEER measurements, diarrhea scores, and weight loss were calculated in GraphPad Prism. The log-rank (Mantel–Cox) test was used for survival analyses. For comparisons of one variable across single-cell clusters between two groups, a Student’s *t*-test was used; for comparisons involving more than two groups, a Wilcoxon rank-sum test was applied. Statistical significance is indicated as follows: *p < 0.05; **p < 0.01; ***p < 0.001; and ****p < 0.0001. Differential gene expression was calculated using the Wilcoxon rank-sum test with Bonferroni correction. Specific statistical tests for each comparison are indicated in the figure legends.

## Results

### Neutrophils aggravate *C. difficile* toxin-induced epithelial injury

**Figure 1.**
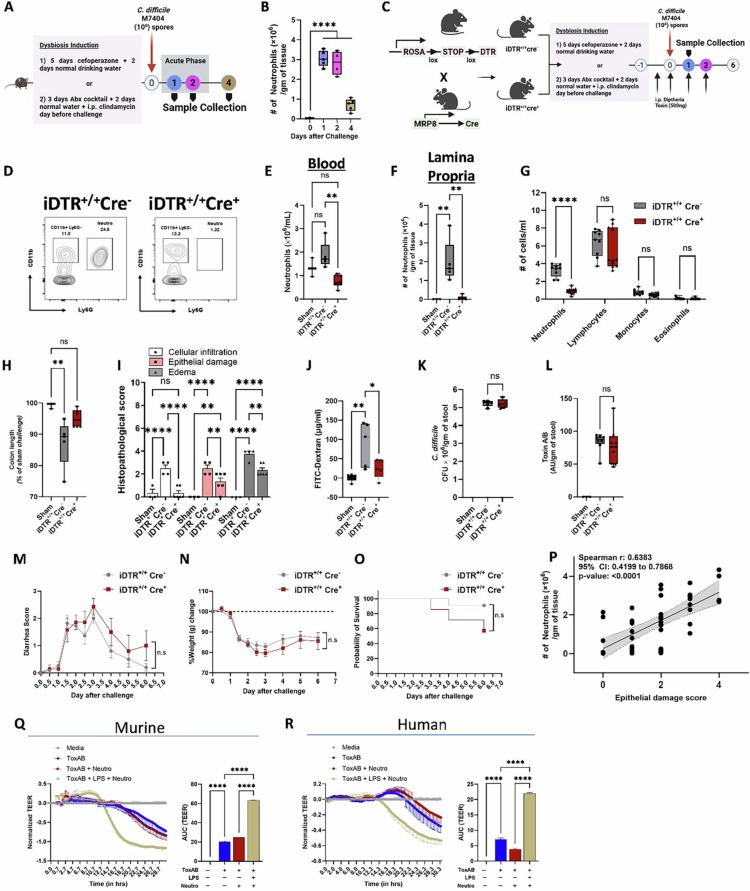
Neutrophils aggravate *C. difficile* toxin-induced epithelial damage. (A) Experimental plan: Age- and gender-matched WT C57BL/6 mice were pre-treated with antibiotics (cefoperazone alone or antibiotic cocktail method) and challenged with 1 × 10^6^ *C. difficile* (M7404) spores by oro-gastric gavage. Animals were euthanized on days 0, 1, 2, and 4, and samples were collected for further analysis. (B) Number of neutrophils in colonic tissue of uninfected (day 0) and *C. difficile-*infected (day 1, 2, and 4) mice. (C–O) Experimental plan: age- and gender-matched iDTR^+/+^Cre^−^ (control) and iDTR^+/+^Cre^+^ mice were pre-treated with antibiotics and challenged with 1 × 10^6^ *C. difficile* (M7404) spores as above. (C) Schematic diagram showing generation of transgenic mice (iDTR^+/+^Cre^−^ and iDTR^+/+^Cre^+^): mice expressing Cre-inducible simian diphtheria toxin (DT) receptor (DTR) (ROSA26-iDTR) were bred to mice with Cre recombinase under control of a neutrophil-specific MRP8 promoter (MRP8-Cre^+^). DT injection 12 hours prior to and on the day of *C. difficile* challenge was used to deplete endogenous neutrophils. For (D–L), samples were collected 1-d after infection. For (M–O), mice were examined daily for 6 d. (D) Representative flow cytometry plots showing neutrophil depletion in iDTR^+/+^Cre^−^ (control) and iDTR^+/+^Cre^+^ mice. (E) Number of neutrophils (×10^6^) per ml of blood, and (F) per gram of colonic tissue; (G) differential cell count (neutrophils, lymphocytes, monocytes, and eosinophils) in blood using Hemavet; (H) colon length, (I) histological scoring of colon sections, (J) serum FITC-dextran, (K) pathogen burden, and (L) toxin titers, from samples collected 1 d after infection. (M–O) Clinical disease progression was monitored during the course of CDI; (M) diarrhea score, (N) weight loss, and (O) survival, over a period of 6 d. (P) Spearman correlation analysis of colonic neutrophil counts and epithelial damage score 1 d after CDI. (Q and R) Change in transepithelial electrical resistance (TEER) and area under the curve TEER plots of (Q) CMT-93 cell monolayers incubated with *C. difficile* toxins A and B (5 ng/ml each), LPS (1 µg/ml), and murine bone marrow neutrophils; and (R) Caco-2 cell monolayers incubated with *C. difficile* toxins A and B (5 ng/ml each), LPS (1 µg/ml), and human peripheral blood neutrophils. For panels (B, E, F, H–L), data shown as box-and-whisker plots (boxes represent the interquartile range, whiskers extend to the minimum and maximum, and lines denote the median); *n* = 3–6 per group; representative of 2–3 independent experiments. For panel (G), data shown as box-and-whisker plots (boxes represent the interquartile range, whiskers extend to the minimum and maximum, and lines denote the median); *n* = 9–11 per group; representative of one experiment. For panel (I), data are shown as mean ± SEM; *n* = 3–6 per group; representative of two independent experiments. For panel (M–N), data are shown as mean ± SEM, for panel (O), data are shown as a survival plot; for (M–O), *n* = 7–11 per group; representative of one experiment. For panel (P), *n* = 33 (data combined from five experiments). For panels (Q–R), curves are plotted as the normalized mean value of three replicates: mouse studies are representative of two independent experiments, and human studies are representative of three independent experiments. Stats: 1-way ANOVA with Tukey’s multiple comparisons (B, E, F, H, J–L, Q, R); two-way ANOVA with Bonferroni's correction (G, I); unpaired *t*-test with Welch’s correction (for AUC of diarrhea score and weight loss; (M and N); Log-rank (Mantel–Cox) test (O); Spearman correlation (P); *p < 0.05; **p < 0.01; ***p < 0.001; and ****p < 0.0001.

In patients with CDI, excessive colonic neutrophilia correlates with worse histopathology and adverse clinical outcomes.^68-70^ In a pre-clinical mouse model (Figure 1A; Supp Figure 1), acute CDI results in colonic neutrophil accumulation followed by a decrease in their numbers during the recovery phase (i.e., by day four after challenge) (Figure 1B). To directly examine the effect of neutrophils on CDI-induced IEC damage, we used transgenic mice that allow for the selective depletion of endogenous neutrophils.^21^ Mice expressing Cre-inducible simian diphtheria toxin (DT) receptor (DTR) (ROSA26-iDTR) were bred to mice with Cre recombinase under control of a neutrophil-specific MRP8 promoter (MRP8-Cre^+^) to generate iDTR^+/+^Cre^+^ and iDTR^+/+^Cre^−^ mice (Figure 1C). DT treatment 12 hours prior to and on the day of infection reduced blood and colon neutrophils in iDTR^+/+^Cre^+^ mice to numbers similar to sham-challenged mice during the acute phase of CDI, while iDTR^+/+^Cre^−^ control mice still had significant neutrophilia (Figure 1C–F). However, DT treatment did not affect the number of lymphocytes, monocytes, and eosinophils in blood on day 1 (Figure 1G). During acute CDI, iDTR^+/+^Cre^+^ mice had less colonic damage as evidenced by longer colon length (Figure 1H) and improved histology scores, including reduced cellular infiltration, edema, and epithelial damage (Figure 1I). The impact of CDI-induced neutrophilia on IEC barrier function was further examined by a FITC-dextran (FD4) permeability assay: 18 hours after CDI, DT-treated iDTR^+/+^Cre^+^ and iDTR^+/+^Cre^−^ mice were fasted for 4 hours and then gavaged with FD4. Plasma samples were collected 6 hours later: we found that the amount of FD4 fluorescence detected in plasma was lower in iDTR^+/+^Cre^+^ mice compared to iDTR^+/+^Cre^−^ (Figure 1J). Despite an effect on IEC barrier, neutrophil depletion during this acute phase of infection did not impact pathogen burden or toxin titers between the two groups (Figure 1K,L). To test the impact of neutrophil depletion on CDI disease progression and outcome, we challenged iDTR^+/+^Cre^−^ and iDTR^+/+^Cre^+^ mice with M7404 spores. Both groups received DT by intraperitoneal injection at the time of infection and 2 additional injections at 24-hour intervals. Depletion of neutrophils did not have a significant impact on clinical disease symptoms, such as diarrhea and weight loss, or on survival (Figure 1M–O). Further supporting our observations, we found that the number of neutrophils per gram of colonic tissue in WT mice with acute CDI exhibited a significant correlation with epithelial damage score (Figure 1P).

To directly test if neutrophils can exacerbate *C. difficile* toxin-induced barrier damage, we co-cultured mouse bone marrow neutrophils with a mouse epithelial cell line (CMT-93) in the presence or absence of *C. difficile* toxins A and B (Figure 1Q and Supp Figure 2A). Compared to untreated controls, incubation of IECs with toxins alone decreased transepithelial electrical resistance (TEER), but addition of bone marrow neutrophils did not alter the toxin-induced drop in TEER (Figure 1Q and Supp Figure 2A). When IECs were incubated with toxins and bone marrow neutrophils in the presence of lipopolysaccharide (LPS), there was a significantly greater reduction in TEER (Figure 1Q and Supp Figure 2A). To determine how these findings could potentially translate to CDI patients, we co-cultured human peripheral blood neutrophils from healthy donors with a human epithelial cell line (Caco-2) ± *C. difficile* toxins and LPS. Somewhat different from mouse data, we did observe a drop in TEER when IECs were exposed to neutrophils + *C. difficile* toxins, but these instances were variable and dependent on the donor (Supp Figure 2B). However, when IECs were exposed to a combination of LPS + neutrophils + *C. difficile* toxins, barrier integrity worsened consistently as compared to all other conditions (Figure 1R and Supp Figure 2B).

### Infiltrating colonic neutrophils exhibit a hyperinflammatory phenotype during CDI

**Figure 2.**
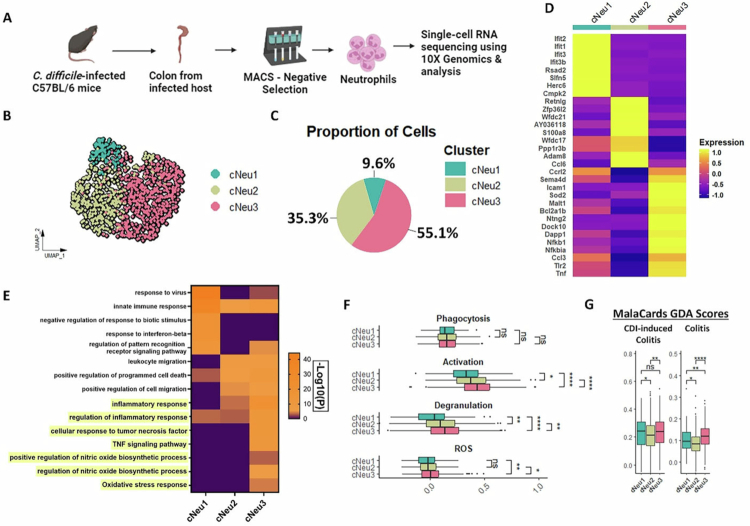
Single-cell transcriptomics reveal an increase in a hyperactivated neutrophil cluster in response to CDI. (A) Experimental plan: Colon tissue was collected from a mouse challenged with *C. difficile* (1 × 10^6^ M7404 spores) during the acute phase of infection (i.e., 2 d after challenge). Neutrophils were enriched using MACS negative selection and submitted for 10× genomics. (B) Uniform Manifold Approximation and Projection (UMAP) of colon neutrophil clusters. (C) Percent distribution of colonic neutrophil clusters. (D) Heatmap of differentially expressed genes for each cluster. (E) Differentially expressed genes from each colon neutrophil cluster of the single-cell dataset were used as the input for Metascape pathway enrichment analysis. (F and G) Module score of colon neutrophils for (F) genes associated with phagocytosis, neutrophil activation, degranulation, and ROS production, and G) genes associated with human CDI and colitis. For (F), gene lists were obtained from prior literature and Gene Ontology database (*Xie et al. 2020* and the Gene Ontology Browser on Mouse Genome Informatics website: https://www.informatics.jax.org/mgihome/GO/project.shtml). For (G), human gene-disease association lists were obtained from MalaCards: *Clostridium difficile* colitis (DOID:0060185) and Colitis (DOID:0060180). For box plots, the middle line represents median values of gene expression, the upper and lower hinges of box represent first and third quartile (25th and 75th percentiles), and the upper/lower whiskers extend from the hinge to the largest/smallest value no further than 1.5*interquartile range (IQR) from the hinge (where IQR is the distance between the first and third quartiles). Stats: two-tailed Wilcoxon rank-sum test (F and G); *p < 0.05; **p < 0.01; ***p < 0.001; and ****p < 0.0001.

Neutrophil traits and phenotypic diversity are influenced by their response to pathogenic insults and tissue microenvironments.^15^^,^^16^^,^^18^^,^^19^ To define the mechanisms underlying neutrophil-induced IEC damage in CDI, we characterized colonic lamina propria neutrophils during the acute phase of infection using single-cell transcriptomics (Figure 2A). High-quality sequencing data were obtained from 1001 colonic neutrophils from a *C. difficile*-infected mouse 2 d after challenge (Figure 2A and Supp Figure 3A–K). Using Uniform Manifold Approximation and Projection (UMAP) and clustering, we identified three transcriptionally distinct colon neutrophil (cNeu) clusters—cNeu1, cNeu2, and cNeu3—with cNeu3 being the most abundant (Figure 2B,C). Differential gene expression (DGE) analyses of these populations revealed increased expression of *Rsad2*, *Ifit1,* and *Ifit2* transcripts in cNeu1; *Retnlg, Ccl6,* and *S100a8* transcripts in cNeu2; and *Nfkbia*, *Nfkb1,* and *Malt1* transcripts in cNeu3 (Figure 2D and Supp Table 3). To better understand the functional state of each population, we performed pathway enrichment analysis and found that cNeu3 displayed a transcriptional program characterized by enrichment of genes related to TNF signaling, inflammatory response, nitric oxide biosynthesis, and oxidative stress responses—hallmarks of an enhanced effector state (Figure 2E).^5^^,^^18^ Given our earlier observation that neutrophils exacerbate epithelial barrier damage without effects on *C. difficile* burden (Figure 1), we scored neutrophils for gene modules associated with phagocytosis, degranulation, activation, and ROS production to determine if these effector states were transcriptionally primed to mediate tissue injury or for other neutrophil functions (e.g., control pathogens via enhanced phagocytosis). While phagocytosis scores were similar across all three clusters, cNeu3 neutrophils exhibited significantly elevated scores for activation, degranulation, and ROS production – consistent with a pro-inflammatory phenotype linked to tissue injury (Figure 2F).^71^^,^^72^ Notably, many genes enriched in cNeu3 were predicted targets of *Nfkb1*, implicating this transcription factor as a potential regulator of the pro-inflammatory and effector programs active in this population (Supp Figure 3L). We sought to determine whether the transcriptional programs defining cNeu3 overlap with gene signatures reported in human intestinal inflammatory diseases. We scored the cNeu clusters for gene signatures upregulated in *C. difficile*-induced colitis and other forms of colitis (gene lists curated by MalaCards,^73^ and found that cNeu3 exhibited enhanced expression of genes linked to both conditions (Figure 2G).

### CDI drives systemic neutrophil transcriptomic alterations that contribute to tissue injury

**Figure 3.**
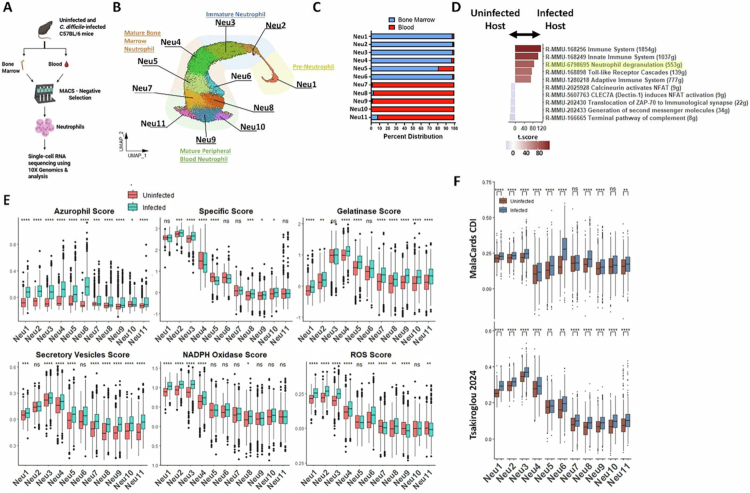
CDI drives systemic neutrophil transcriptomic alterations that contribute to tissue injury. (A) Experimental plan: Bone marrow and blood samples were collected from an uninfected mouse and a mouse challenged with *C. difficile* (1 × 10^6^ M7404 spores) on day 2 after infection. Neutrophils were enriched using MACS negative selection and submitted for 10× genomics. (B) UMAP of bone marrow and blood neutrophil clusters. (C) Percent distribution of bone marrow and blood neutrophil clusters. (D) Waterfall plot of gene set enrichment analysis of bone marrow and blood neutrophils: comparison between data from uninfected and infected mouse. (E and F) Module scoring of bone marrow and blood neutrophils from uninfected and infected mouse for genes associated with (E) granule protein, NADPH oxidase formation, and ROS production; and (F) with human CDI. For (E), gene lists were used from the Reactome database, and for (F), gene lists were curated from MalaCards and from *Tsakiroglou et al. 2024*. For box plots, the middle line represents median values of gene expression, the upper and lower hinges of box represent first and third quartile (25th and 75th percentiles), and the upper/lower whiskers extend from the hinge to the largest/smallest value no further than 1.5*interquartile range (IQR) from the hinge (where IQR is the distance between the first and third quartiles). Stats: two-tailed Wilcoxon rank-sum test (E and F); *p < 0.05; **p < 0.01; ***p < 0.001; and ****p < 0.0001.

To define CDI-induced changes in neutrophil heterogeneity at the site of their development and maturation (i.e., bone marrow) and subsequent mobilization (i.e., blood), and to determine whether CDI also induces shifts in neutrophil cellular states beyond canonical maturation stages, we examined the transcriptomic profile of bone marrow and blood neutrophils at homeostasis and during acute infection (Figure 3A and Supp Figure 3M–O). We used previously established methodology, including Clustree and differential gene expression to annotate the neutrophils (Supp Figure 4 and Supp Table 4).^74^^,^^75^ Altogether, we identified 11 neutrophil cellular states across bone marrow and blood, which aligned closely with previously reported maturation stages: Neu1 resembled pre-neutrophils, Neu2–3 resembled immature neutrophils, and Neu4–11 corresponded to mature bone marrow and blood neutrophil populations (Figure 3B,C).^15^^,^^18^^,^^62^

We performed a gene set enrichment analysis using Reactome^76^ to define the impact of CDI on fundamental cellular pathways of these cells. We found that genes associated with degranulation were among the top 3 that were upregulated in neutrophils from the *C. difficile*-infected host (Figure 3D). In addition, CDI broadly increased the expression of genes associated with the generation of azurophil, gelatinase, and secretory granule proteins, and ROS production (Figure 3E). In particular, expression of specific granule proteins was enhanced in immature (Neu3) and peripheral blood (Neu8–11) neutrophils following infection (Figure 3E). Lastly, we investigated whether *C. difficile*-induced transcriptional changes in murine bone marrow and blood neutrophils reflect those observed in human CDI. Both bone marrow and blood neutrophils upregulate genes associated with CDI (curated by MalaCards and DEGs identified in PBMCs from CDI patients) (Figure 3F).^63^ Together, these data suggest that CDI primes bone marrow and blood neutrophils for enhanced pro-inflammatory activity and cytotoxic potential before tissue infiltration, paralleling transcriptional changes seen in human GI pathology.

### Clusters with cNeu3 signatures are present in bone marrow and blood during acute CDI

**Figure 4.**
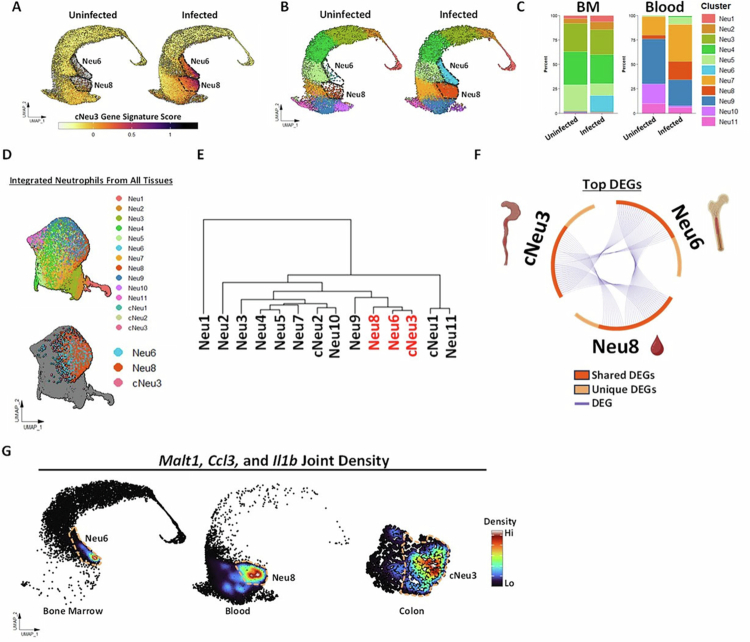
Clusters with cNeu3 signatures are present in bone marrow and blood during acute CDI. (A) Feature plot displaying the module score of cNeu3 signature genes in bone marrow and blood neutrophils from uninfected and infected mice. (B) UMAP of bone marrow and blood neutrophil clusters from uninfected and infected mouse with Neu6 and Neu8 highlighted in a black dotted line showing a significant increase in these clusters during CDI. (C) Percent distribution of bone marrow and blood clusters in the corresponding tissues of uninfected and infected mouse. (D) Sequencing data from bone marrow, blood, and colon neutrophils of both the uninfected and infected mouse was re-integrated in Seurat using tissue compartment as the integration variable, and two UMAPs were created: one displaying all neutrophils and another showing only the Neu6, Neu8, and cNeu3 clusters. (E) Dendrogram to show the relation of neutrophil clusters based on average gene expression. (F) Circos plot of top 50 DEGs of Neu6, Neu8, and cNeu3 calculated in each tissue separately (Neu6 in bone marrow, Neu8 in blood, and cNeu3 in colon). Each arc represents a gene list, with individual genes positioned along the arc. Dark orange indicates genes shared across multiple lists, while light orange marks genes unique to a single list. Purple lines represent differentially expressed genes that are shared across multiple gene lists. (G) Joint density maps for *Malt1*, *Ccl3*, and *Il1b* were generated using Nebulosa (via ScPubR) for bone marrow, blood, and colon neutrophils of the infected host, with Neu6, Neu8, and cNeu3 boundaries indicated by yellow dotted lines. Density values are overlaid on the UMAP embedding.

Pathological perturbations are known to drive stress-driven/emergency granulopoiesis, resulting in generation of neutrophils to meet the demands of countering an infectious agent.^77^ However, in certain contexts, infection alters the number of neutrophils at different stages of development and/or creates new sources of heterogeneity that diverge from the homeostatic developmental trajectory.^19^^,^^20^ To determine if similar cells—particularly the hyperactive cNeu3 cluster—are present in other tissue compartments, we scored bone marrow and blood neutrophils for expression of top DEGs that characterize cNeu3 (Figure 4A). Module scoring revealed that two clusters in bone marrow and blood (Neu6 and Neu8, respectively) were highly enriched for the cNeu3 gene signature, and compared to the uninfected mouse, the proportion of these two neutrophil clusters expanded significantly after CDI (Figure 4B,C). Data analysis by a complementary approach (i.e., by integrating bone marrow, blood, and colon neutrophil clusters) resulted in Neu6, Neu8, and cNeu3 coalescing into one unified cluster (Figure 4D). Further, hierarchical clustering based on average gene expression revealed a high degree of transcriptomic similarities between Neu6, Neu8, and cNeu3 despite differences in tissue compartment (Figure 4E). Compared to other neutrophils, Neu6, Neu8, and cNeu3 shared numerous top 50 DEGs (identified by DGE analysis of clusters in their respective tissue separately) and could be identified by co-expression of *Malt1, Ccl3,* and *Il1b* (Figure 4F,G).

### cNeu3-like neutrophil activation state is associated with epithelial damage during CDI

**Figure 5.**
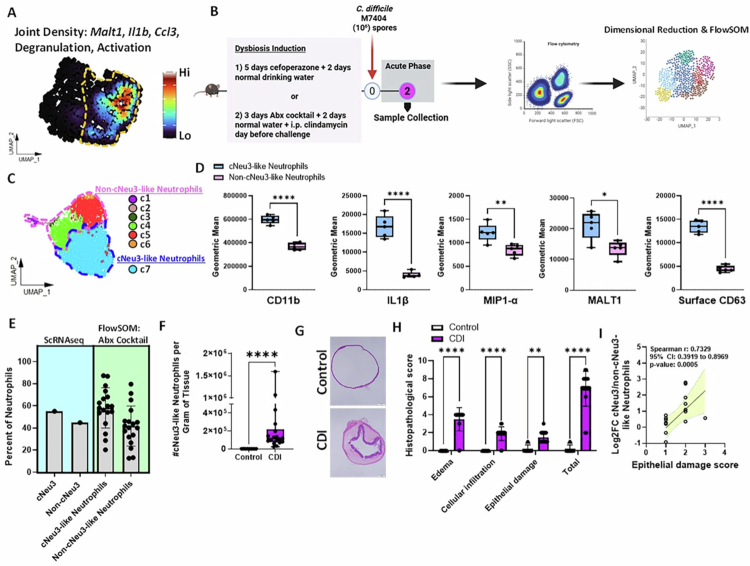
cNeu3-like neutrophil activation state is associated with epithelial damage during CDI. (A) UMAPs showing the expression of *Malt1*, *Ccl3*, *Il1b*, and neutrophil degranulation and activation module scores across cNeu populations, with cNeu3 outlined by a yellow dotted line. Joint densities for these features were computed using Nebulosa (via ScPubR) and overlaid onto the UMAP embedding. (B) Experimental plan: Colon tissue was collected from WT mice challenged with *C. difficile* (1 × 10^6^ M7404 spores) on day 2 after infection. Lamina propria neutrophils were stained with flow cytometry antibodies to cell surface and intracellular markers (predicted to mark cNeu3 cells in our transcriptomics dataset). Dimensional reduction and FlowSOM clustering were performed on neutrophils. (C) UMAP of clusters identified by FlowSOM with cNeu3-like neutrophils (blue) and non-cNeu3-like neutrophils (pink) outlined in dotted lines. (D) Geometric mean fluorescent intensities (gMFIs) of CD11b, IL-1β, MIP1-α, MALT1, and surface CD63. (E) Proportion of cNeu3 and non-cNeu3 neutrophils detected with single-cell transcriptomics vs. FlowSOM (antibiotic cocktail-induced dysbiosis model) during infection. (F) Number of cNeu3-like neutrophils per gram colon tissue. (G) Representative H&E stained cecal sections from uninfected controls and mice with CDI and (H) histological scoring of tissue specimens. (I) Spearman correlation analysis of Log_2_FC of the number of cNeu3-like to non-cNeu3-like neutrophils plotted against the respective epithelial damage score. Panels are presented as box-and-whisker plots (boxes represent the interquartile range, whiskers extend to the minimum and maximum, and lines denote the median). Panel (D) shows data from 1 experiment; representative of three independent experiments; *n* = 4–8 per group. Panels (E–I) show data combined from three independent experiments, total *n* = 8 (uninfected control) and 18 (CDI). Stats: unpaired *t*-test with Welch’s correction (D–H); Mann–Whitney test (F), two-way ANOVA with Bonferroni's correction (H), and Spearman correlation (I); *p < 0.05; **p < 0.01; ***p < 0.001; and ****p < 0.0001.

To determine whether the transcriptionally defined cNeu3 state corresponds to a distinct activation phenotype *in vivo*, we performed flow cytometric analysis of colonic neutrophils during acute CDI. In our single-cell dataset, cNeu3 cells were characterized by increased expression of *Malt1*, *Ccl3*, and *Il1b*, along with genes associated with neutrophil activation and degranulation (Figure 5A). Guided by this transcriptional signature, we selected corresponding proteins to include: (i) MALT1, CCL3 (MIP-1α), IL-1β; (ii) CD11b (surrogate for activated neutrophils)^78^; and (iii) surface CD63 (surrogate for degranulation)^79^ as markers to identify the cNeu3 phenotype by flow cytometry (experimental design shown in Figure 5B; gating strategy of neutrophils in Supp Figure 5A).

After optimizing the staining protocols for multi-color flow cytometry, we performed high-dimensional analysis of live CD11b⁺Ly6G⁺ neutrophils using UMAP for dimensional reduction and FlowSOM for unsupervised clustering. Clusters exhibiting high CD63 expression together with elevated IL-1β, CD11b, MALT1, and MIP-1α were classified as cNeu3-like (Figure 5C,D and Supp Figure 5B–C). As a complementary approach, we also defined the presence of cNeu3-like neutrophils in our data by using a conventional flow cytometry gating strategy. In this case, we found that cells that had high expression of CD63, IL-1β, and CD11b (Supp Figure 5D) also had high MALT1 and MIP-1α expression (Figure 5D and Supp Figure 5D–F). Together, these data provide a flow cytometric validation of the transcriptionally defined cNeu3 state. Similar to the scRNAseq data, FlowSOM analysis of colon samples showed that cNeu3-like cells were proportionally more abundant than non-cNeu3-like neutrophils during infection (Figure 5E). This relative enrichment was observed across dysbiosis models induced by either an antibiotic cocktail (Figure 5E) or cefoperazone treatment (Supp Figure 5G). Compared to uninfected mice, the absolute number of cNeu3-like neutrophils increased in the colon of mice with CDI (Figure 5F).

As expected, CDI resulted in significant epithelial damage and changes in colonic histopathology compared with uninfected control mice (Figure 5G and H). To assess the relationship between the presence of inflammatory neutrophil states and IEC injury, we calculated log_2_ fold change of cNeu3-like cells relative to non-cNeu3-like neutrophils (both identified via flow cytometry) for each mouse. We found a strong correlation between the proportion of cNeu3-like cells and IEC damage—a higher percentage of these cells was associated with increased epithelial damage score (Figure 5I).

### TNF treatment of neutrophils induces cNeu3 phenotype *in vitro*

**Figure 6.**
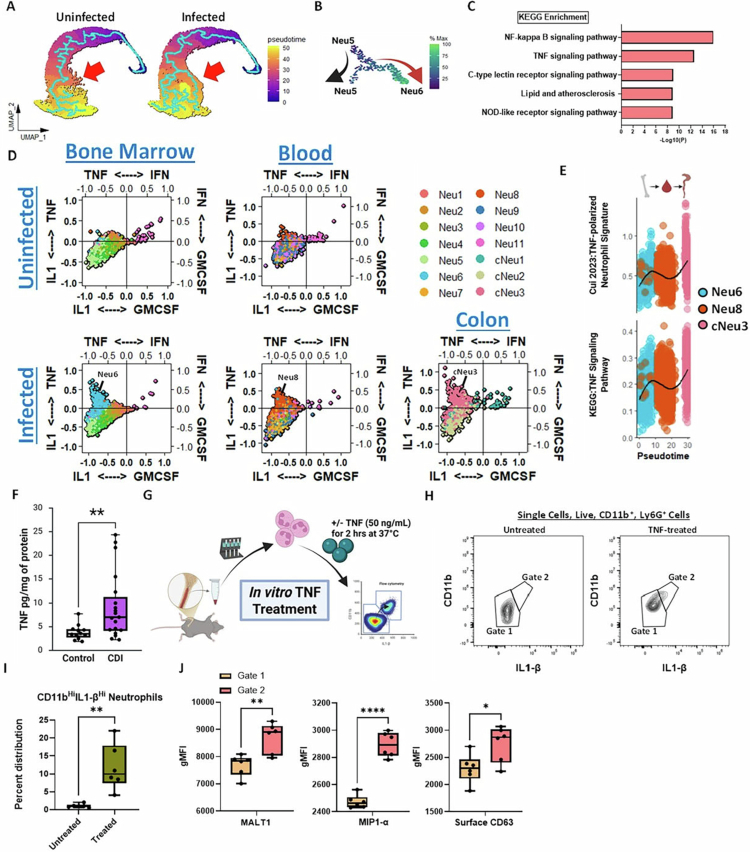
cNeu3-like neutrophils share a transcriptomic profile with TNF-polarized neutrophils during acute CDI, and TNF treatment induces this phenotype *in vitro*. (A) Monocle pseudotime trajectory analysis of bone marrow and blood neutrophil clusters from uninfected and infected mouse. (B) Cell fate analysis performed using Monocle3 by analyzing genes upregulated as cells transition from Neu5 to Neu6 along the pseudotime trajectory. Each point represents a cell in UMAP space, and cells are colored by the gene module upregulated as cells transition from Neu5 to Neu6. (C) Metascape KEGG pathway enrichment analysis of genes upregulated as cells transition from Neu5 to Neu6. (D) Neutrophil cytokine-induced polarization cellular state plots of bone marrow, blood, and colon neutrophils from uninfected and *C. difficile*-infected mouse. DEGs from neutrophils polarized with TNF, type I/II interferons, IL-1α/β, or GM-CSF were utilized to define neutrophil transcriptomic states induced by the corresponding cytokines. For this analysis, gene lists were obtained from Cui et al. 2023. (E) *Cui 2023* TNF-polarized neutrophil gene signature and KEGG TNF signaling pathway signature for Neu6, Neu8, and cNeu3 plotted against pseudotime. (F) Amount of TNF (pg/mg of protein) collected from uninfected and *C. difficile*-infected mice during acute CDI (i.e., day 2 after challenge). (G) Experimental plan: Neutrophils were isolated from the bone marrow of WT mice and enriched by MACS bead-based negative selection. Neutrophils were plated at 1 × 10^6^ cells/well in 96-well plates and then treated with recombinant murine TNF (50 ng/ml) or control (PBS) for 2  h. Cells were incubated at 37 °C and then stained for flow cytometry. (H) Representative flow cytometry plot showing CD11b and IL-1β staining in untreated and TNF-treated samples. (I) Percent distribution of CD11b^hi^IL-1β^hi^ in untreated and TNF-treated samples. (J) Comparison of MALT1, MIP1α, and CD63 gMFI in gate 1 vs. gate 2 populations in untreated and TNF-treated samples. Panels (F) and (J and K) are presented as box-and-whisker plots (boxes = interquartile range, whiskers = minimum and maximum, line = median). Panel (F) shows data combined from 4 independent experiments, *n* = 14 (control) and 18 (CDI) (F); panels (H) and (J) are representative of 2 independent experiments, *n* = 3 per group; panel I is combined data points from two independent experiments, total *n* = 6 per group. Stats: Mann–Whitney test (F); Spearman correlation (G); unpaired *t*-test with Welch's correction (I and J); *p < 0.05; **p < 0.01; ***p < 0.001; and ****p < 0.0001.

To further explore the development of Neu6, Neu8, and cNeu3, pseudo-temporal ordering and trajectory analysis was performed on single-cell transcriptomics data of the uninfected and *C. difficile*-infected host (Figure 6A). Monocle pseudotime analysis revealed that neutrophil differentiation in an uninfected host proceeds as a single trajectory with branches diverging at the level of Neu7 (Figure 6A). However, during CDI, the neutrophil developmental trajectory bifurcates earlier (at the level of Neu5), and cells either developmentally progress as they did in the uninfected host or transition to Neu6 and Neu8 cellular states (Figure 6A). To characterize factors involved in cell fate decisions leading to Neu6 development, we calculated genes upregulated as cells transition from Neu5 to Neu6 and found increased gene expression associated with NF-κB and TNF signaling (Figure 6B and C).

Host- and pathogen-derived products augment the neutrophil transcriptome, leading to enhanced effector functions and pro-inflammatory capacity.^80-82^ Since there was a dramatic infection-induced increase in Neu6 and Neu8, we examined the possibility that these neutrophils are a cellular state that develops in response to elevated levels of CDI-induced pro-inflammatory cytokines. Recently, *Cui* and colleagues used single-cell transcriptomics to develop a “dictionary of immune responses to cytokines” in which they characterized the transcriptome of immune cells polarized with 86 cytokines.^82^ They revealed that IL-1α/β, type I/II interferons, TNF, and GM-CSF each induced distinct transcriptional states in neutrophils, defining four major polarization programs.^82^ Utilizing four variable cellular state plots, we found that Neu6, Neu8, and cNeu3 indeed differentially expressed genes induced by TNF, but not for other cytokines (Figure 6D). Corroborating these findings, an immune response enrichment analysis (IREA) predicted that the DEGs of Neu6, Neu8, and cNeu3 were a result of enhanced TNF signaling (Supp Figure 6A).

Further supporting TNF-driven neutrophil programming, TNF signaling genes increased with pseudotime as cells advanced toward the site of infection (Figure 6E).^82^ Concurrently with this genetic signature, we found an increase in colonic tissue TNF levels during the acute phase of CDI (i.e., day 2 after infection; Figure 6F). To test if TNF signaling can shift neutrophils towards a cNeu3-like state, we cultured bone marrow neutrophils with recombinant TNF and assessed its impact on various markers used to define cNeu3 (Figure 6H–J and Supp Figure 6B). We found that TNF treatment *in vitro* did recapitulate the cNeu3-like phenotype that was seen *in vivo*, as evidenced by increased proportion of CD11b^hi^IL-1β^hi^ neutrophils in the TNF-treated conditions and a higher mean fluorescent intensity of MALT1, MIP-1α, and CD63 in these cells (Figure 6I,J). Together, these data support a scenario whereby *C. difficile*-induced TNF alters neutrophil developmental trajectory towards a hyperactivated state.

### Activating human neutrophils with TNF increases damage to *C. difficile* toxin-treated IECs

**Figure 7.**
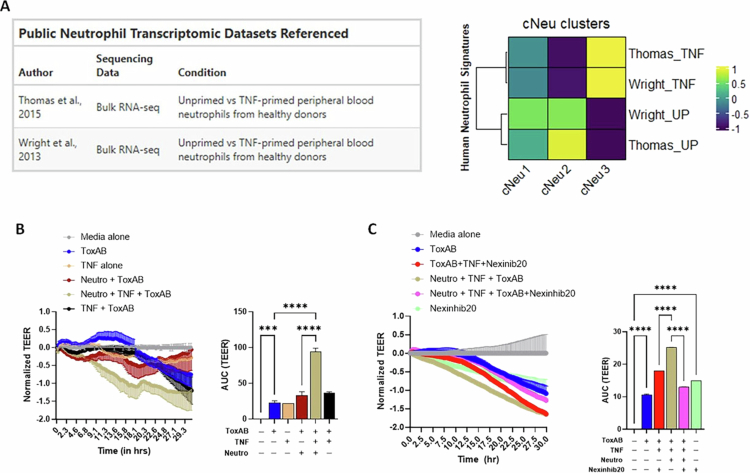
TNF-primed neutrophils worsen *C. difficile* toxin-induced IEC damage. (A) Human TNF-primed neutrophil gene signature scoring of colon neutrophil clusters. Public bulk RNA-seq datasets^60^^,^^61^ were re-analyzed to identify the top 50 genes differentially expressed between unprimed and TNF-primed human neutrophils. This gene set was applied to mouse colon neutrophils, and scores are shown as a heatmap. (B and C) Change in TEER and area under curve TEER plots of Caco_2_ cell monolayers treated with combinations of (B) toxins A/B (5 ng/ml each), TNF (20 ng/ml), and human neutrophils; and (C) toxins A/B (5 ng/ml each), TNF (20 ng/ml), human neutrophils, and Nexinhib20 (10 µM). Curves are plotted as the normalized mean value of three replicates representative of *n* = 5 donors for B, and *n* = 3 donors for C. Stats: one-way ANOVA (B and C); *p < 0.05; **p < 0.01; ***p < 0.001; and ****p < 0.0001.

Recent evidence indicates that neutrophils share a core inflammatory transcriptional program across humans and mice, with conserved responses to diverse activating stimuli.^83^ Our results showed a prominent TNF-induced gene signature in cNeu3 neutrophils. In order to test if this gene signature aligns with reported TNF-driven programs in human neutrophils, we compared our dataset with two published bulk RNA-seq datasets in which human peripheral blood neutrophils were treated with TNF.^60^^,^^61^ We found that gene modules corresponding to the published data from TNF-primed human neutrophil groups were indeed most upregulated in our cNeu3 population (Figure 7A). Overall, these analyses support the presence of a core TNF-driven pathway that is conserved in cNeu3 neutrophils.

To directly test if TNF-activated neutrophils exacerbate *C. difficile* toxin-induced epithelial damage, we performed co-culture experiments using human neutrophils from healthy donors and Caco-2 monolayers exposed to combinations of TNF, *C. difficile* toxins, and neutrophils. As reported previously,^83^ TNF exposure alone reduced IEC barrier integrity (Figure 7B and Supp Figure 6C). Exposure of IECs to *C. difficile* toxins, either alone or in combination with TNF or neutrophils, also resulted in TEER drop, but the magnitude of this effect was variable depending on the donor (Figure 7B and Supp Figure 6C). However, across all donors, we found that the highest drop in TEER was observed when IECs were exposed to *C. difficile* toxins + TNF + neutrophils (Figure 7B). Together, these data indicate that TNF-activated neutrophils can exacerbate barrier damage induced by *C. difficile* toxins and TNF.

Our transcriptomic atlas and proteomic data suggest that the cNeu3 cellular state is marked by enhanced degranulation, particularly azurophil granule release, as indicated by increased surface CD63. To test whether TNF-activated neutrophils exacerbate IEC permeability through azurophil granule release, we utilized Nexinhib20, which blocks Rab27a–JFC1 binding and impairs trafficking and release of azurophilic granules without affecting other processes such as phagocytosis or NETosis.^46^ Consistent with Figure 7B, the largest decrease in TEER occurred when IECs were exposed to *C. difficile* toxins + TNF + neutrophils, which was significantly greater than that observed with *C. difficile* toxins either alone or in combination with TNF + Nexinhib20 (Figure 7C and Supp Figure 6D). Although Nexinhib20 alone resulted in a reduction in TEER, addition of Nexinhib20 to IECs exposed to TNF + *C. difficile* toxins + neutrophils significantly improved barrier integrity, with TEER values returning to levels similar to those observed in toxin-treated IECs alone (Figure 7C and Supp Figure 6D).

## Discussion

Neutrophils are critical effector cells for controlling infection. However, dysregulation of this arm of immunity leads to bystander tissue injury, which contributes to disease.^2-5^ In patients with *C. difficile*-induced colitis, white blood cell count (mainly comprising neutrophils) >15 × 10^9^/L is commonly used to predict disease severity, and a high number of colonic tissue neutrophils is associated with worse CDI outcomes.^12^^,^^84^^,^^85^ Despite correlative evidence suggesting a critical role for neutrophils in worsening CDI, direct experimental evidence of their deleterious effects has been lacking. Here, we used multiple complementary approaches to conduct an in-depth investigation of the role of neutrophils in CDI. The most important findings of our study are: (i) neutrophils exert pathogenic functions which worsen *C. difficile* toxin-induced intestinal barrier integrity *in vitro* and *in vivo*; (ii) utilizing scRNAseq, we established the first single-cell transcriptomic atlas of neutrophils in CDI and detected increased gene expression associated with production of granule protein genes, NADPH oxidase, ROS, pro-inflammatory cytokines, and pathways associated with neutrophil-derived tissue damage; (iii) a CDI-induced neutrophil cluster, cNeu3, was detected in each tissue sample by scRNAseq and elevated numbers of cNeu3-like neutrophils were detected in the colon of *C. difficile*-challenged mice; (iv) increased proportion of cNeu3-like neutrophils was associated with enhanced epithelial damage in mice during acute CDI; (v) cNeu3-like neutrophils share a transcriptomic profile with TNF-polarized neutrophils; (vi) TNF treatment induces this phenotype *in vitro*; and (vii) TNF-exposed human neutrophils worsen toxin-induced IEC damage *in vitro*, and an inhibitor of neutrophil exocytosis ameliorated this damage.

Multiple studies have clearly shown that in CDI, markers of inflammation (including neutrophil-associated responses) are better correlated with disease severity than pathogen burden, but the majority of current treatments are pathogen-directed.^10^^,^^11^^,^^86^^,^^87^ Thus, neutrophils or neutrophil-mediated responses are attractive targets for development of novel therapies to improve outcomes for this deadly infection. However, neutrophils have a dichotomous role in CDI: increased number of peripheral blood and colon neutrophils are pathogenic and correlate with worse disease,^12^^,^^63^^,^^86^ but complete abrogation of neutrophils in animal models is also associated with worse survival.^88^^,^^89^ Therefore, it is critical to define both the beneficial and pathogenic neutrophil subtypes, effector functions, and neutrophil-derived molecules in CDI. Our study is a significant attempt to address this key knowledge gap. Using unbiased single-cell transcriptomics, we found that all neutrophils in the *C. difficile*-infected host upregulated genes associated with immune effector functions, but more specifically, we detected a neutrophil state, cNeu3, which upregulated genes associated with tissue injury and human disease. Although we didn’t have multiple biological replicates for the transcriptomics data, the key findings were, in fact, validated at the protein level using spectral flow cytometry across many biological replicates. Further, high-dimensional flow cytometry identified the cNeu3-like neutrophils in colonic tissue, and we show that these cells are associated with enhanced IEC damage.

High levels of circulating TNF is associated with increased risk of 90-d mortality in CDI patients.^11^ In this study, our transcriptomics analyses and *in vitro* experiments begin to reveal some potential mechanisms by which this inflammatory cytokine can contribute to CDI pathogenesis. We found that the hyperactivated cNeu3 cluster aligned with TNF-primed neutrophils, and *in vitro* TNF treatment was sufficient to change the phenotype of bone marrow neutrophils towards cNeu3-like neutrophils. TNF activates canonical NF-κB signaling in neutrophils, a pathway linked to hyperactivation in severe infections.^72^^,^^90^ Consistent with this, *Nfkb1* emerged as a top candidate transcription factor regulating genes enriched in cNeu3.^91^ cNeu3/cNeu3-like neutrophils also upregulated *Malt1*/MALT1, a component of the CARD9–BCL10–MALT1 signalosome that promotes NF-κB-dependent transcription.^92^ Notably, cNeu3-like neutrophils also upregulated *Ccl3*, a gene induced by TNF in both humans and mice.^60^^,^^61^ *Ccl3* encodes the chemokine MIP-1α, which drives leukocyte recruitment and amplifies local inflammation.^93^^,^^94^ Here, we show that cNeu3-like neutrophils express significantly higher MIP-1α *in vivo* compared with non-cNeu3-like neutrophils, and this phenotype is recapitulated with TNF treatment *in vitro*. In prior reports, MIP-1α-CCR1 signaling has been shown to mediate toxin A-induced neutrophil recruitment and epithelial injury, and disruption of this pathway via genetic deletion reduces neutrophil infiltration, MPO activity, and epithelial damage.^94^^,^^95^ Together, these findings support a scenario in which TNF-NF-κB axis can promote a hyperactivated, degranulating neutrophil state that drives MIP-1α-dependent neutrophil recruitment and contributes to IEC barrier injury.

TNF, released by immune and non-immune cells during infection, is a potent driver of neutrophil priming and activation.^96^^,^^97^ One consequence of this activation is TNF-induced degranulation via p38 MAPK-dependent actin remodeling and PLC-mediated calcium flux, facilitating granule docking and fusion.^43^^,^^98-101^ cNeu3-like neutrophils exhibited increased surface CD63 expression, a marker of azurophilic granule degranulation, suggesting active degranulation. Exocytosis of azurophilic granule proteins mediates tissue injury via proteolysis, oxidative stress, and disruption of epithelial barrier function.^5^^,^^102^ Trafficking of azurophilic granules requires actin remodeling and Rab27a–JFC1–mediated translocation,^46^^,^^103^ and pharmacologic inhibition of this interaction using Nexinhib20 suppresses neutrophil degranulation *in vivo.*^46^ Building on this mechanistic evidence, we applied Nexinhib20 to our TNF-treated human neutrophil–IEC co-culture system and found that this inhibitor of azurophilic granule exocytosis markedly reduced neutrophil-induced TEER defects, restoring barrier integrity to levels similar to toxin-treated monolayers alone. Notably, Nexinhib20 alone reduced TEER, suggesting potential off-target or epithelial-intrinsic effects that warrant further investigation. Collectively, these findings indicate that TNF-driven neutrophil hyperactivation primes azurophilic degranulation, linking inflammatory signaling to tissue injury and highlighting neutrophil granule exocytosis as a potential therapeutic target in CDI.

In this study, we used a model of diphtheria toxin–inducible depletion of endogenous neutrophils. Our data using this model demonstrate that neutrophils directly contribute to impaired barrier function during acute CDI *in vivo*. There was no impact of neutrophil depletion on *C. difficile* burden or toxin titers, and we did not see any impact of neutrophil depletion on diarrhea score, weight loss, or survival. Our findings are consistent with prior work in which: (i) reduced colonic neutrophil infiltration by CD18 blockade in rabbits^104^ or anti-MIP-2 in rats^105^ was associated with decreased intestinal permeability; and (ii) antibody-mediated neutrophil depletion in mice did not result in any significant survival advantage after CDI or any effect on *C. difficile* pathogen burden.^88^^,^^106^ However, our data does differ from some other studies that reported higher mortality in neutrophil-depleted mice with CDI.^88^^,^^89^ In these studies, increased translocation of gut bacteria into deeper tissues was observed and proposed to contribute to disease severity and mortality.^88^^,^^89^ While the exact reason for the discrepant findings between our data and some published studies remains unknown, factors such as differences in methods of neutrophil depletion—published studies used antibody-mediated depletion vs. our study used a diphtheria toxin-mediated model—could contribute to these differences. Importantly, such discordant findings highlight the multifaceted role of neutrophils in CDI pathogenesis, whereby they can impact both barrier damage and/or bacterial translocation. These observations also raise a key point: while neutrophils and neutrophil-mediated responses represent attractive therapeutic targets, such a strategy must be optimized, as broad targeting of all neutrophils could have unexpected and unintended side effects. We posit that more precise strategies that focus on specific neutrophil states, their functions, or neutrophil-derived molecules are likely to offer safer and more effective therapeutic avenues in the future.

While our study provides mechanistic insights into TNF-associated neutrophil hyperactivation during *C. difficile* infection, several avenues remain for future investigation. Longitudinal studies assessing cNeu3 dynamics throughout infection and recurrent CDI, alongside clinical parameters such as weight loss, fecal consistency (diarrhea scores), will define the temporal behavior and clinical relevance of this transient neutrophil state. Additionally, targeted inhibition of specific granule proteins *in vitro* and *in vivo* could pinpoint the mediators responsible for epithelial barrier disruption and identify potential therapeutic targets. Although our findings suggest that TNF blockade could reduce CDI-induced hyperactivated neutrophil populations, it is important to note that TNF is a pleiotropic cytokine affecting multiple biological pathways and cell types; therefore, its global inhibition could have wide-ranging effects. In fact, somewhat contrary to our observations, anti-TNF therapy in mice results in worsened histopathology.^107^^,^^108^ Therefore, in order to successfully use the currently available FDA-approved anti-TNF therapies,^46^^,^^109^ it will be important to define the impact of both global (e.g., via TNF blockers) and/or targeted (e.g., via neutrophil-specific TNFR deletion) TNF inhibition on examining phenotypic shifts toward the cNeu3-like state and subsequent impact on gut barrier permeability using animal models and relevant human primary cells (e.g., human neutrophils co-cultured with enteroids and organoids).

Overall, our data suggest that *C. difficile*-induced TNF alters the neutrophil transcriptome, resulting in the expansion of a hyperactivated neutrophil cluster, and that inhibition of TNF-driven neutrophil polarizing pathways, such as priming/activation or degranulation, may prove to be an effective therapeutic target for infectious or inflammatory diseases, such as severe CDI.

## Supplementary Material

Supplemental Material Supplemental_file.docx

Supplementary Tables.xlsx Supplementary Tables.xlsx

## Data Availability

All sequencing data generated in this study have been submitted at NCBI’s Gene Expression Omnibus (GEO) repository under the GEO Series accession number GSE299936. The published data used in this study were retrieved from the GEO (accession numbers GSE109467,^62^ GSE70068,^61^ GSE40548,^60^ and GSE276395.^63^
