# Supplementary material for: Phenotypic profiling of neutrophils in acute Clostridioides difficile infection identifies a TNF-induced activation signature associated with epithelial damage
Source: Gut Microbes. 2026 Apr 23;18(1):2659430. doi: 10.1080/19490976.2026.2659430 (PMC13108352; doi:10.1080/19490976.2026.2659430)
Supplement: Supplemental Material — Supplemental_file.docx [file KGMI_A_2659430_SM2339.docx]

**Supplemental**

**Supplementary Figures**

**
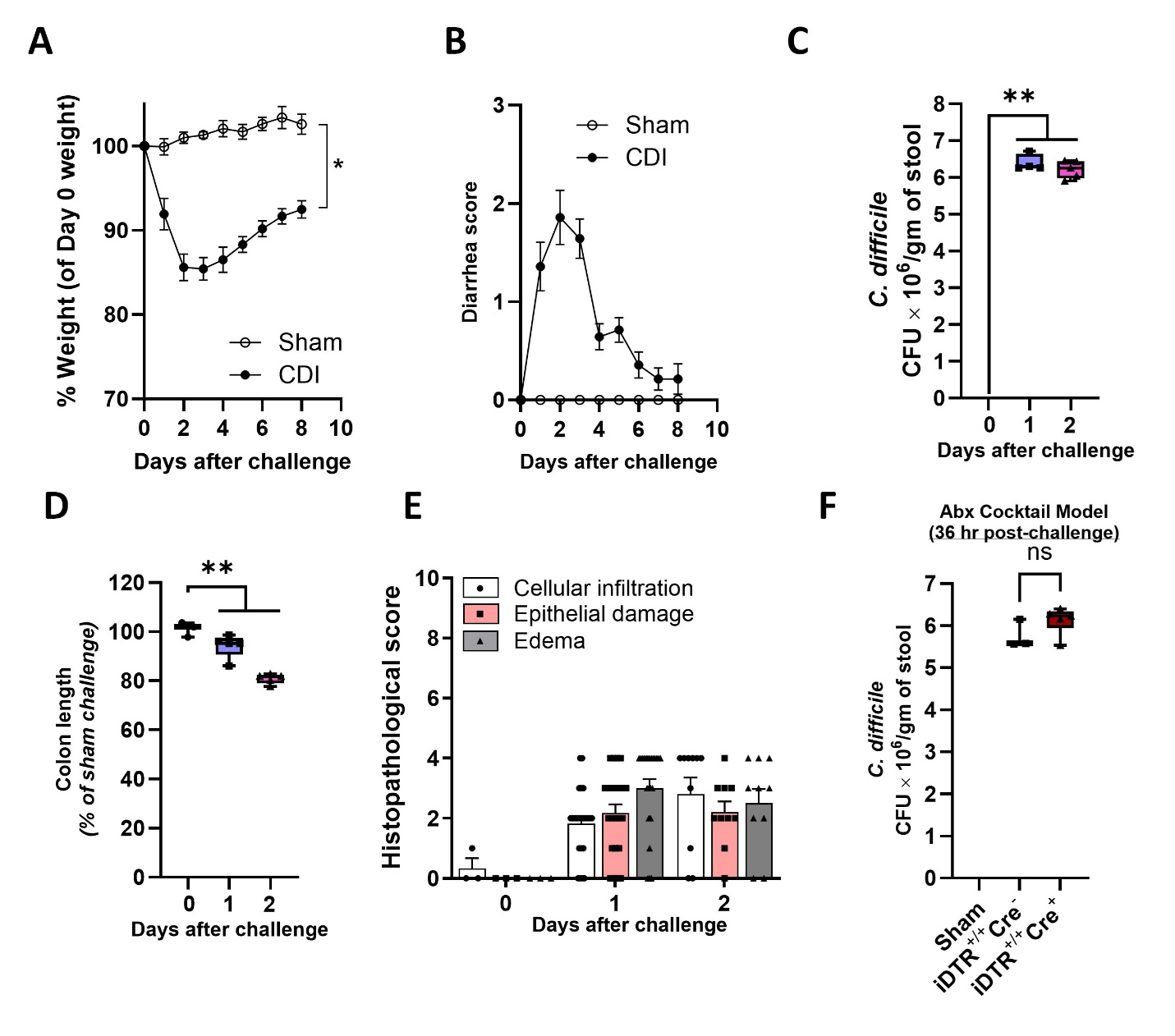
**

**Supplementary Figure 1: Murine model of CDI.** Experimental plan: Age- and gender-matched WT C57BL/6 mice were pre-treated with antibiotics for 5 days in drinking water and challenged with 1x10^6^ *C. difficile* (M7404) spores by oro-gastric gavage 2 days after cessation of antibiotics. Shown here is **A)** percent weight loss; **B)** diarrhea score of sham and *C. difficile*-challenged mice; and **C)** CFUs/gram stool (cefoperazone model); **D)** colon length; **E)** histopathology score on day 0, day 1, and 2 of infection; and **F)** CFUs/gram stool (antibiotic cocktail model). Data shown as mean ± SEM; N = 3-6 per group; representative of 2 independent experiments. Stats: 1-way ANOVA (A-B); 2-tailed unpaired Student’s t-test (C-F); *p < 0.05; **p < 0.01; ***p < 0.001; ****p < 0.0001.


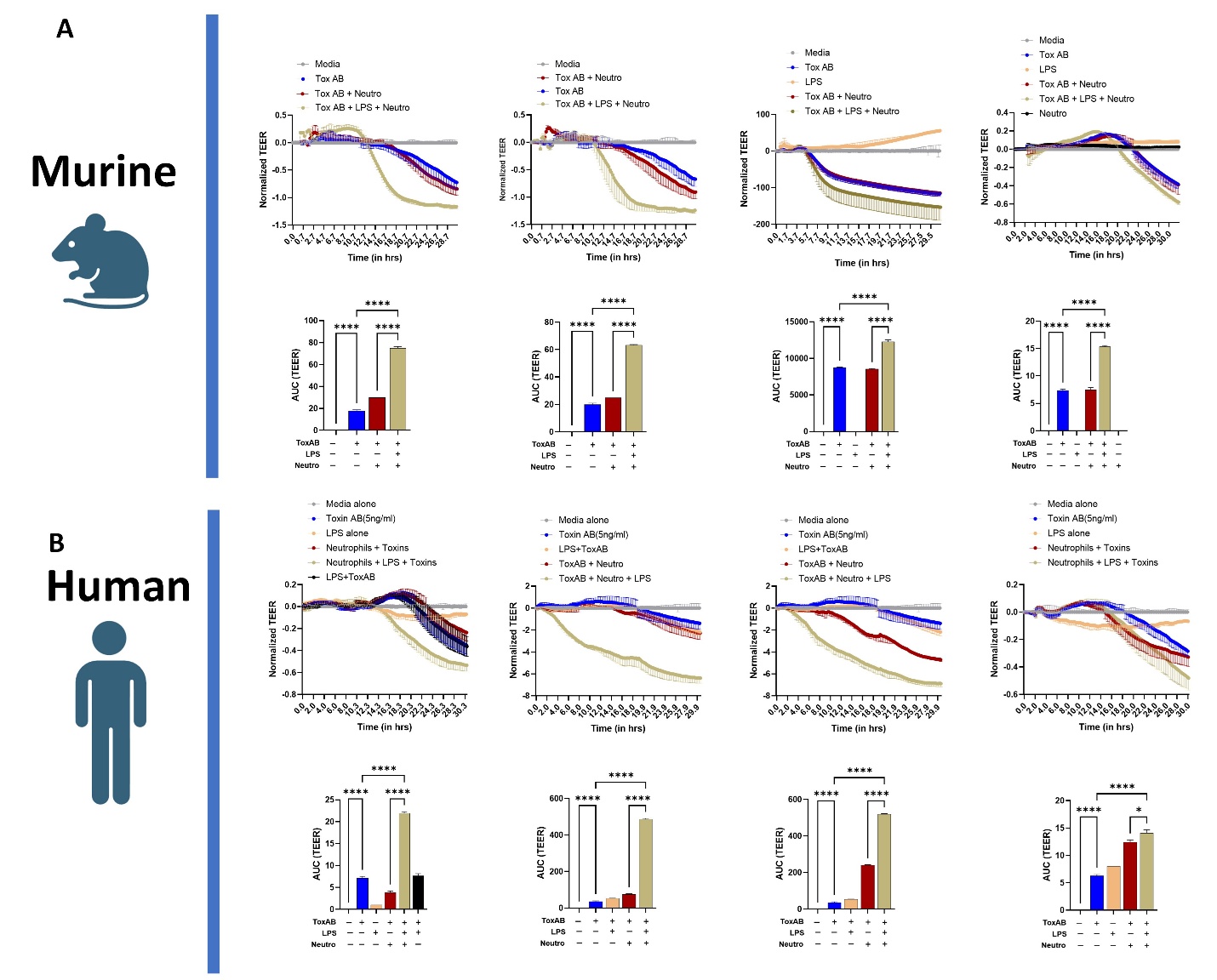


**Supplementary Figure 2: Murine and human neutrophils worsen *C. difficile* toxin-induced IEC permeability.** Change in transepithelial electrical resistance (TEER) and area under curve TEER plots of **A)** CMT-93 cell monolayers incubated with *C. difficile* toxins A and B (5ng/ml each), LPS (1µg/ml), and murine bone marrow neutrophils; and **B)** Caco2 cell monolayers incubated with *C. difficile* toxins A and B (5ng/ml each), LPS (1µg/ml), and human peripheral blood neutrophils. Shown here is data from all individual experiments. Stats: 1-way ANOVA; *p < 0.05; **p < 0.01; ***p < 0.001; ****p < 0.0001.


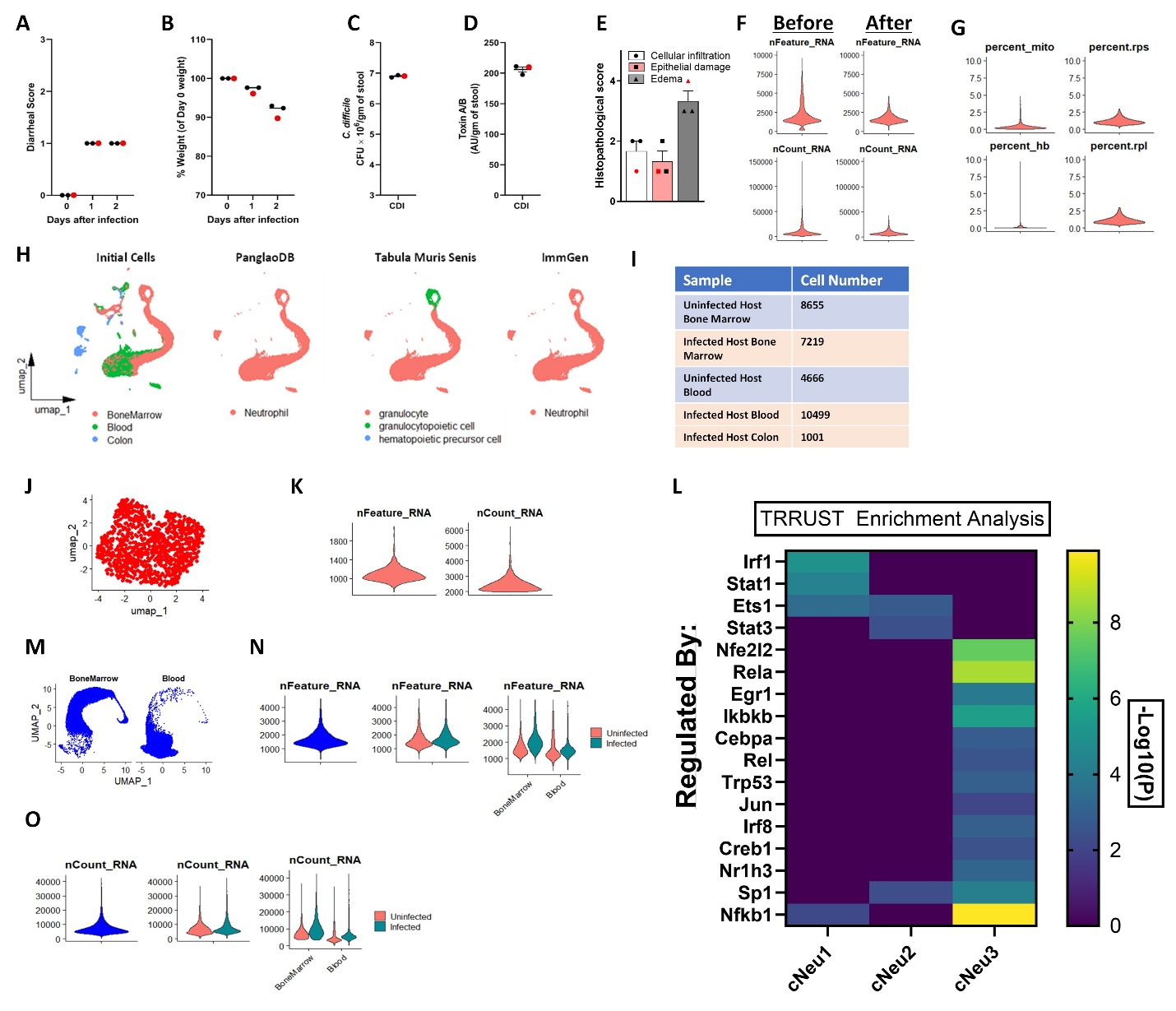


**Supplementary Figure 3: Neutrophil distributions after CDI and clinical disease score of the *C. difficile*-infected mouse used for neutrophil single-cell transcriptomics.** Experimental plan: Three male WT C57BL/6 mice were pre-treated with antibiotics for 5 days in drinking water and challenged with 1x10^6^ *C. difficile* (M7404) spores by oro-gastric gavage 2 days after cessation of antibiotics. Samples were collected 2 days after infection and submitted for 10X genomics sequencing. Here we show **A)** diarrhea score, **B)** percent body weight, **C)** pathogen burden, **D)** cecal toxin level, and **E)** cecal histopathology score of all three mice. The mouse whose bone marrow, blood, and colon sample was used for sequencing is highlighted in red **(A-D).** **F)** Violin plots of cells before and after filtering out low quality cells (i.e., cells below 1% nFeatures_RNA) and doublets (i.e., cells above 92.3% nFeatures_RNA & cells filtered by scDblFinder). **G)** Violin plots of cells after filtering out cells with mitochondrial genes > 5%, hemoglobin genes > 10%, and ribosomal genes > 3%. **H)** UMAP of initial cell clustering, UMAP of cells enriched for neutrophil genes (termed “Neutrophil” from PanglaoDb) utilizing the AUCell package, UMAP of cells labeled as cell types from the *Tabula Muris Senis* and ImmGen datasets using the SingleR package. **I)** Cell number distribution of neutrophils from each sample utilized for downstream analyses. **J)** UMAP of colon neutrophils and **K)** violin plots displaying nFeature_RNA and nCount_RNA counts. **L)** TRRUST Enrichment analysis performed through Metascape on colon neutrophil DEGs. **M)** UMAP of bone marrow and blood neutrophils split by tissue. **N)** nFeature_RNA counts for the integrated Seurat object, split by infection, and split by infection and tissue. **O)** nCount_RNA counts for the integrated Seurat object, split by infection, and split by infection and tissue.


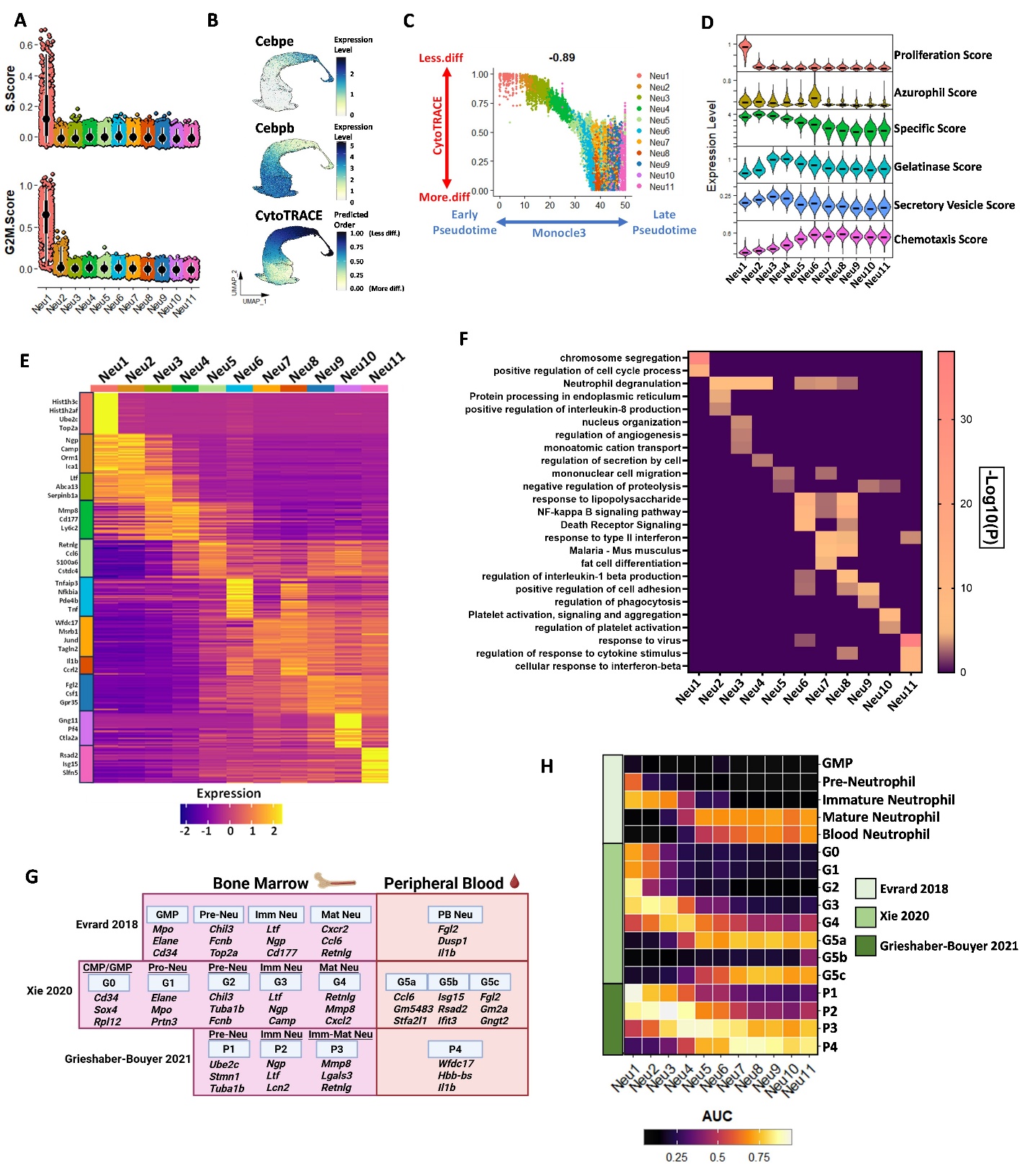


**Supplementary Figure 4: Single-cell atlas of bone marrow and blood neutrophil states at homeostasis and during CDI. A)** Cell cycle scores of neutrophil clusters (S-phase progression and G2/M-phase progression). **B)** Feature plots of markers upregulated during early stages (*Cebpe*) and late stages (*Cebpb*) of neutrophil development. CytoTrace analysis was performed to place neutrophil clusters on spectrum of less differentiated to most differentiated. **C)** Feature scatter plot of predicted cell ordering by CytoTrace and Monocle3. Each point in the scatter plot represents a cell (colored by cluster). Pearson correlations between the two features are displayed above the plot. **D)** Module scores of genes involved in neutrophil proliferation, granule protein (azurophil, specific, gelatinase, and secretory vesicles) production, and chemotaxis. **E)** Heatmap displaying < 50 differentially expressed genes for each cluster, with signature genes highlighted on the left. **F)** DGE analysis was utilized as the input of Metascape pathway enrichment analysis and top pathways were plotted in a heatmap. **G)** Top 3 signature DEGs of previously characterized mouse neutrophil populations in bone marrow and blood at steady state (*15, 18*). **H)** Signature DEGs were used to score neutrophil clusters from uninfected and *C. difficile*-infected mice and plotted in a heatmap as AUC values.

**
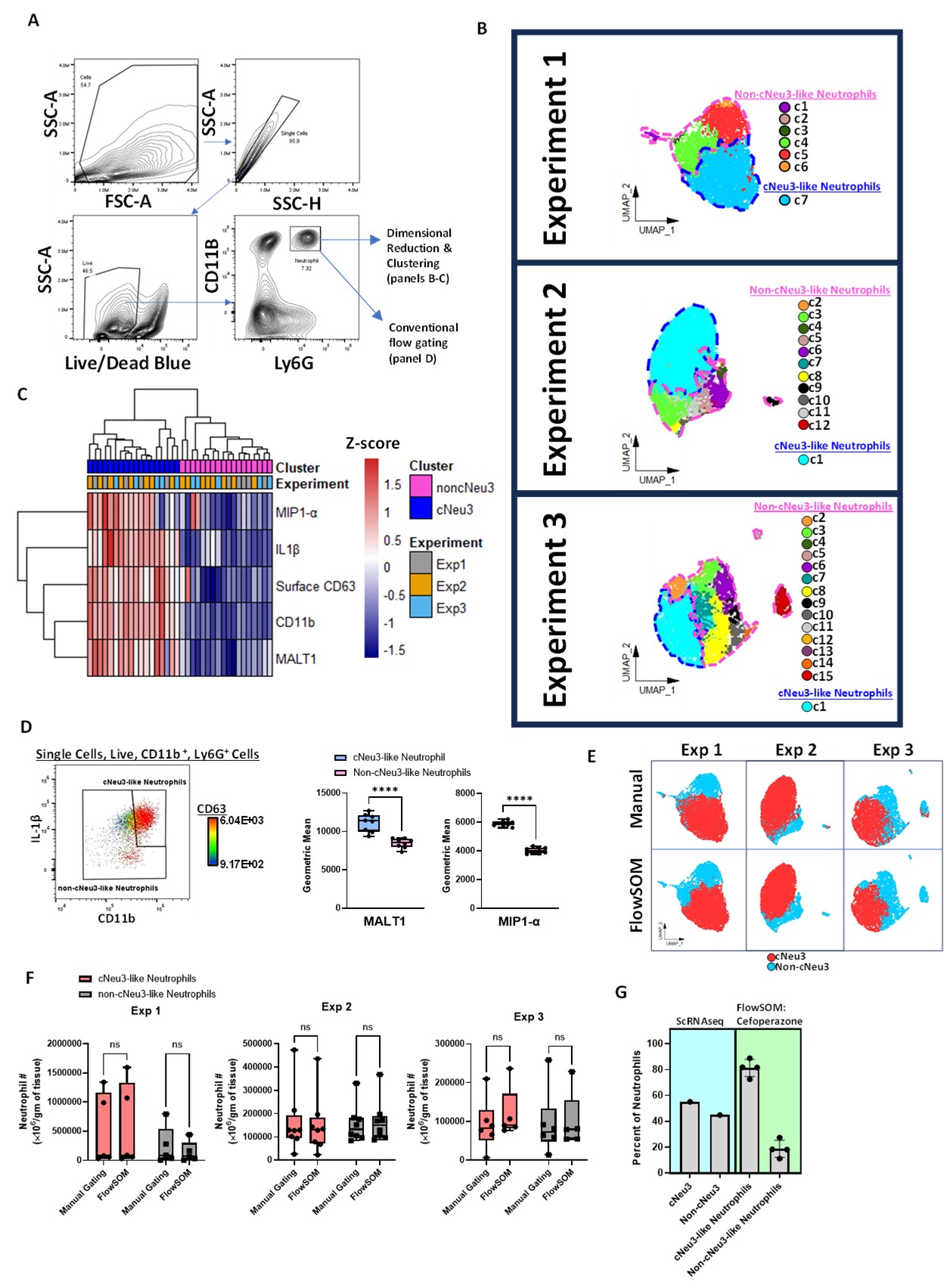
**

**Supplementary Figure 5: Flow cytometric analysis of cNeu3-like neutrophils *in vivo*. A)** Neutrophil gating strategy for *in vivo* CDI experiments. **B)** UMAP of clusters identified by FlowSOM for 3 independent experiments with cNeu3-like neutrophils (blue) and non-cNeu3-like neutrophils (pink) outlined in dotted lines. **C)** Z-scores of CD11b, IL-1β, MIP1-α, MALT1, and surface CD63 gMFIs were calculated independently for each experiment and shown for cNeu3-like and non–cNeu3-like neutrophils in a combined heatmap. **D)** Gating strategy for cNeu3-like neutrophils and non-cNeu3-like neutrophils and gMFIs for MALT1 and MIP1-α. **E)** UMAPs of cNeu3-like neutrophils and non-cNeu3-like neutrophils identified by manual gating and by FlowSOM for each experiment. **F)** Comparison of cNeu3-like neutrophils and non-cNeu3-like neutrophils identified by manual gating and by FlowSOM for each experiment. **G)** Proportion of cNeu3 and non-cNeu3 neutrophils detected with single-cell transcriptomics vs FlowSOM (cefoperazone dysbiosis model). Panels are presented as box-and-whisker plots (boxes represent the interquartile range, whiskers extend to the minimum and maximum, and lines denote the median). Panels B, E, and F display data collected from 3 independent experiments and plotted separately, total n = 14 (uninfected control) and 18 (CDI). Panel C shows data combined from 3 independent experiments, total n = 14 (uninfected control) and 18 (CDI). Panel D shows data from 1 experiment; representative of 3 independent experiments; n = 4-8 per group. Panel G is representative of one experiment n=4 mice with CDI. Stats: unpaired t-test with Welch’s correction (D); 2-way ANOVA with Bonferroni’s correction (F); *p < 0.05; **p < 0.01; ***p < 0.001; ****p < 0.0001.

**
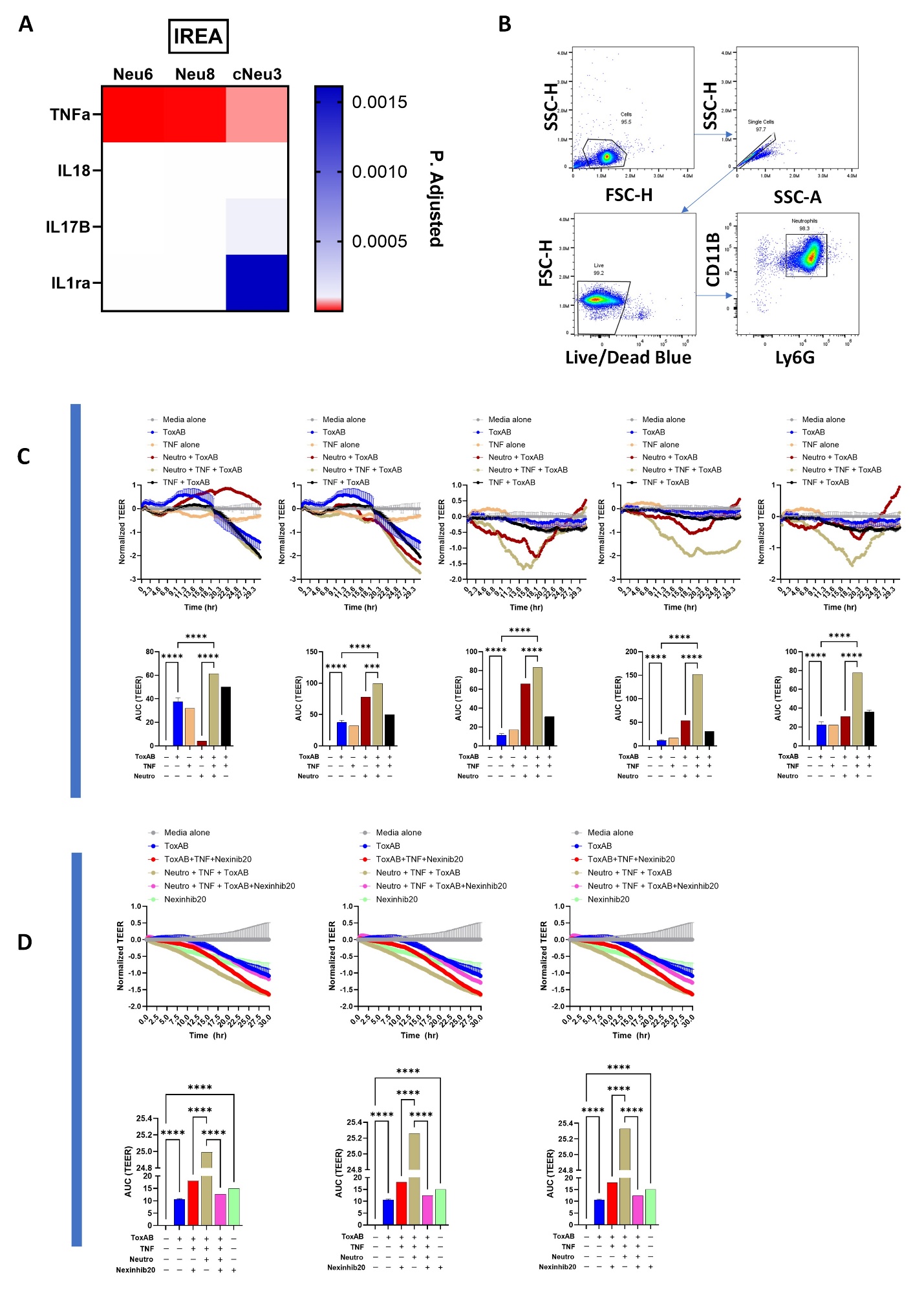
**

**Supplementary Figure 6: Effect of TNF-primed neutrophils and Nexinhib20 on *C. difficile* toxin-induced IEC damage. A)** Immune response enrichment analysis (IREA) performed on DEGs of Neu6, Neu8, and cNeu3. **B)** Neutrophil gating strategy for *in vitro* murine neutrophil experiments. Change in TEER and area under curve TEER plots of Caco2 cell monolayers treated with combinations of **C)** toxins A/B (5ng/ml each), TNF (20ng/ml), and human neutrophils; and **D)** toxins A/B (5ng/ml each), TNF (20ng/ml), human neutrophils and Nexinhib20 (10µM). Shown here are normalized TEER curves of each donor plotted independently. Stats: 1-way ANOVA; *p < 0.05; **p < 0.01; ***p < 0.001; ****p < 0.0001.

**List of Supplementary Tables**

1. **Supplementary Table 1: Histology Scoring Parameters**
2. **Supplementary Table 2: Gene Lists for Module Scoring**
3. **Supplementary Table 3: DGE Analysis of Colon Neutrophil Clusters**
4. **Supplementary Table 4: DGE Analysis of BM and Blood Neutrophil Clusters**

**Supplemental Methods**

**Post-Sequencing, Filtering, and Quality Control**

Cell Ranger 7.0.0 (https://www.10xgenomics.com/) was utilized for sequencing read alignment to the mm10 mouse transcriptome, quantification of the expression of cellular transcripts, and generating a matrix file with expression counts for each sample, with genes as rows and cell Unique Molecular Identifier (UMI) as columns. HDF5 file formats were produced and utilized for analysis in Seurat using the Read10X_h5 function (*111*). In Seurat, data was merged and filtered. Cells below 1% nFeatures_RNA (poor quality cells) and above 92.3% (based on predicted multiplet rate of recovered cells) were excluded from analyses (*111, 112*). Dead/dying cells were filtered out based on presence of >5% mitochondrial genes. Additionally, cells with high hemoglobin genes (>5%) and high ribosomal genes (>3%) were filtered out. Doublets were further filtered out of the dataset using scDblFinder (*113*). Following initial filtering, the standard Seurat analysis of integrated data was utilized. We split the merged object by infection status to create a list (SplitObject), normalized (NormalizeData) and identified variable features (FindVariableFeatures) for each dataset independently, selected variable features (SelectIntegrationFeatures) across datasets for integration, identified anchors (FindIntegrationAnchors), used anchors to integrate the datasets (IntegrateData), scaled the data (ScaleData), ran PCA (RunPCA) followed by UMAP (RunUMAP), found neighbors (FindNeighbors) at 1:24 dimensions, and lastly found clusters (FindClusters) at 0.5 resolution.

AUCell was utilized to identify cells in our dataset that are enriched in the genes from a neutrophil gene dataset obtained from PanglaoDB database (*49*). Cells enriched for these mouse neutrophil genes were then labeled as either “Neutrophil” or “non_Neutrophil.” Neutrophils obtained from AUCell annotation were used as the input for subsequent annotation processes using SingleR. Neutrophils identified were compared to bone marrow cells in Tabula Muris Senis dataset and neutrophils in ImmGen (*50, 51*). Neutrophil-labeled cells were retained for further analysis.

111 Butler A, Hoffman P, Smibert P, Papalexi E, Satija R. Integrating single-cell transcriptomic data across different conditions, technologies, and species. Nat Biotechnol. 2018;36:411–420. doi: 10.1038/nbt.4096.

112 Stuart T, Butler A, Hoffman P, Hafemeister C, Papalexi E, Mauck WM, 3rd, Hao Y, Stoeckius M, Smibert P, Satija R. Comprehensive integration of single-cell data. Cell. 2019;177:1888–1902e1821. doi: 10.1016/j.cell.2019.05.031.

113 Germain PL, Lun A, Garcia Meixide C, Macnair W, Robinson MD. Doublet identification in single-cell sequencing data using scDblFinder. F1000Res. 2021;10:979. doi: 10.12688/f1000research.73600.1.
